# The role of the aryl hydrocarbon receptor in the development of cells with the molecular and functional characteristics of cancer stem-like cells

**DOI:** 10.1186/s12915-016-0240-y

**Published:** 2016-03-16

**Authors:** Elizabeth A. Stanford, Zhongyan Wang, Olga Novikov, Francesca Mulas, Esther Landesman-Bollag, Stefano Monti, Brenden W. Smith, David C. Seldin, George J. Murphy, David H. Sherr

**Affiliations:** Department of Environmental Health, Boston University School of Public Health, 72 East Concord Street (R-408), Boston, Massachusetts 02118 USA; Boston University Molecular and Translational Medicine Program, 72 E. Concord Street, Boston, MA 02118 USA; Department of Medicine, Boston University School of Medicine, Section of Computational Biomedicine, Boston, MA 02118 USA; Department of Medicine, Boston University School of Medicine, Section of Hematology and Oncology, 650 Albany Street, Boston, MA 02118 USA; Boston University and Boston Medical Center, Center for Regenerative Medicine (CReM), 710 Albany Street, Boston, MA 02118 USA

**Keywords:** Aryl hydrocarbon receptor, Breast cancer, Environment, Sox2, Tumor initiating cells

## Abstract

**Background:**

Self-renewing, chemoresistant breast cancer stem cells are believed to contribute significantly to cancer invasion, migration and patient relapse. Therefore, the identification of signaling pathways that regulate the acquisition of stem-like qualities is an important step towards understanding why patients relapse and towards development of novel therapeutics that specifically target cancer stem cell vulnerabilities. Recent studies identified a role for the aryl hydrocarbon receptor (AHR), an environmental carcinogen receptor implicated in cancer initiation, in normal tissue-specific stem cell self-renewal. These studies inspired the hypothesis that the AHR plays a role in the acquisition of cancer stem cell-like qualities.

**Results:**

To test this hypothesis, AHR activity in Hs578T triple negative and SUM149 inflammatory breast cancer cells were modulated with AHR ligands, shRNA or AHR-specific inhibitors, and phenotypic, genomic and functional stem cell-associated characteristics were evaluated. The data demonstrate that (1) ALDH^high^ cells express elevated levels of *Ahr* and *Cyp1b1* and *Cyp1a1*, AHR-driven genes, (2) AHR knockdown reduces ALDH activity by 80 %, (3) AHR hyper-activation with several ligands, including environmental ligands, significantly increases ALDH1 activity, expression of stem cell- and invasion/migration-associated genes, and accelerates cell migration, (4) a significant correlation between *Ahr* or *Cyp1b1* expression (as a surrogate marker for AHR activity) and expression of stem cell- and invasion/migration-associated gene sets is seen with genomic data obtained from 79 human breast cancer cell lines and over 1,850 primary human breast cancers, (5) the AHR interacts directly with *Sox2*, a master regulator of self-renewal; AHR ligands increase this interaction and nuclear SOX2 translocation, (6) AHR knockdown inhibits tumorsphere formation in low adherence conditions, (7) AHR inhibition blocks the rapid migration of ALDH^high^ cells and reduces ALDH^high^ cell chemoresistance, (8) ALDH^high^ cells are highly efficient at initiating tumors in orthotopic xenografts, and (9) AHR knockdown inhibits tumor initiation and reduces tumor *Aldh1a1*, *Sox2*, and *Cyp1b1* expression in vivo.

**Conclusions:**

These data suggest that the AHR plays an important role in development of cells with cancer stem cell-like qualities and that environmental AHR ligands may exacerbate breast cancer by enhancing expression of these properties.

**Electronic supplementary material:**

The online version of this article (doi:10.1186/s12915-016-0240-y) contains supplementary material, which is available to authorized users.

## Background

Given the emerging evidence that common environmental carcinogens play a significant role in cancer [1], increased attention has been paid to molecular mechanisms through which pollutants affect tumor formation, invasion and/or progression [2–4]. Historically, most studies on environmental chemical carcinogenesis centered on the ability of genotoxic chemicals to damage DNA, induce mutations, and initiate cancers [5–7]. However, recent data suggest alternative, non-genotoxic pathways involving cellular receptors that can be activated by environmental ligands. One such receptor is the aryl hydrocarbon receptor (AHR). The AHR is the only ligand-activated member of the Per-ARNT-Sim (bHLH/PAS) family of transcription factors, all of which play important roles as environmental- and physiological stress-sensing proteins [8]. The AHR has been best studied for its ability to be activated by dioxins, polychlorinated biphenyls, and polycyclic aromatic hydrocarbons [9], all of which are high priority chemicals on the U.S. Agency for Toxic Substances and Disease Registry list of pollutants of greatest concern to human health (http://www.atsdr.cdc.gov/SPL/resources).

Ligand-bound AHR induces P450 enzymes such as CYP1B1 and CYP1A1, which are capable of generating mutagenic intermediates. However, more recent work suggests that the AHR, which is expressed at aberrantly high levels and is chronically active in several cancers, plays an ongoing role in tumor progression by enhancing tumor invasion and migration [10–15]. The contribution of the AHR to the later stages of cancer may be mediated by non-genotoxic endogenous ligands, which chronically drive AHR transcriptional activity [16, 17]. Here, it is postulated that environmental ligands mimic this effect and drive cancer progression, at least in part, by increasing the development and/or function of cells exhibiting cancer stem-like cell (CS_L_C) properties.

Recent evidence suggests that invasion and eventual metastasis leading to patient death is mediated, to a disproportionate extent, by chemoresistant, long-lived cancer stem cells, sometimes referred to as tumor-initiating cells [18–25]. Breast cancer stem cells can be defined by (1) expression of genes associated with ‘normal’ tissue stem cells (e.g. *Notch1*,*2*, *Sox2*, *Pou5F1*/*Oct4*) and with invasion and migration (e.g. *Twist1*,*2*, *Vim*, *Snai1, Snai2*) [26–30]; (2) formation of spheroid colonies in ultra-low adherence cultures [31]; (3) elevated levels of aldehyde dehydrogenases (ALDH), enzymes associated with chemoresistance, high histological tumor grade, and poor prognoses [19, 21, 32]; (4) the propensity to self-renew while spawning progenitor cells [31, 33]; and (5) an increased tumor initiation capacity in xenografts [31, 33]. Here, we operationally define ‘breast cancer stem-like cells’ (BCS_L_C) as tumor cells robustly expressing the five aforementioned characteristics in a continuum of ‘stem-ness’ in which some cells are more stem-like than others at any given time. Clearly, identifying factors responsible for the development of cells with cancer stem cell qualities is an important step towards understanding why many patients relapse, even several years after remission.

The AHR plays an important role in tissue-specific embryonic development, hematopoietic stem cell self-renewal, pluripotent stem cell and neural stem cell differentiation, and megakaryocyte/erythroid stem cell growth [34–40]. Here, complementing parameters of ‘stem-ness’, including ALDH enzyme activity, stem cell-, invasion- and migration-associated gene expression, tumorsphere formation, migration rate, chemoresistance, and tumor formation at limiting concentrations in xenografts were assessed to test the hypothesis that the AHR similarly influences development and function of BCS_L_Cs. The potential for the AHR to directly interact with the *Sox2* gene, a master regulator of normal tissue-specific stem cell self-renewal and differentiation, was of particular interest.

These studies were performed primarily with ER^−^/PR^−^/Her2^−^ triple negative breast cancer (TNBC) cell lines: Hs578T, derived from a carcinomosarcoma, and SUM149, derived from an inflammatory breast cancer (IBC). TNBC lines were selected for these studies primarily because no effective targeted therapeutic is yet available for this class of breast cancers and because we wanted to evaluate AHR signaling in the absence of its well-established interactions with the estrogen receptor [41]. Results in those lines were compared with genomic outcomes in 79 breast cancer cell lines and more than 1,850 primary cancers. Our results show that the AHR is involved in the control of phenotypic, genomic, and functional cancer stem cell markers in ER^−^/PR^−^/Her2^−^ cells, strongly implicating an important role for the AHR in acquisition of stem cell-like qualities, encouraging development of AHR-targeted therapeutics, and raising the possibility that environmental AHR ligands may drive BCS_L_C development or activity.

## Results

### AHR expression is elevated in ALDH1^high^ TNBCs

We have previously published data demonstrating elevated expression of transcriptionally (‘constitutively’) active AHR in human breast cancer cell lines [10, 15, 42, 43]. The expression of nuclear AHR in ER^−^/PR^−^/Her2^−^ human breast cancer-derived Hs578T cells and in inflammatory ER^−^/PR^−^/Her2^−^ breast cancer-derived SUM149 cells (Additional file 1: Figure S1A) was consistent with these reports. Furthermore, a predominance of nuclear AHR in primary human breast cancers (Additional file 1: Figure S1B, middle and bottom panels), but not in normal breast tissue (Additional file 1: Figure S1B, top panel), supports the conclusion that the AHR is constitutively active in primary cancers as well. Importantly, non-epithelial cells did not express AHR, normal epithelial cells in ducts had a low level of AHR staining, similar to our previous findings in rats [44], and all AHR staining seen in normal epithelial cells was cytoplasmic, indicating inactive AHR. Note that the stains presented here are representative of similar staining observed in 50 human breast cancer samples fixed on a tissue microarray.

Work from several laboratories indicates a role for the AHR in tissue-specific stem cell development [34–38], suggesting a general role for the AHR in stem cell biology. We and others have demonstrated that the AHR is highly expressed and constitutively active in breast cancers and that its activity correlates with tumor aggressiveness [10, 44–47]. Since cancer stem cells contribute to tumor progression, we postulated that the AHR plays a role in the development of breast cancer cells with stem cell-like characteristics (BCS_L_C).

Several investigators have shown that CD44^+^/CD24^−^ cell staining is not an entirely consistent indicator of tumor initiating ability in ER^−^/PR^−^/Her2^−^ breast cancer cells due to over-staining of TNBCs [23, 48–51]. Over-expression or non-specific staining for these prototypic cancer stem cell markers also precluded their use in our studies (data not shown). Therefore, ALDH activity, which appears to be a more selective functional marker for TNBC stem-like cells [19, 23, 52, 53], was used here for marking of and enriching for cancer stem-like cells.

**Fig. 1:**
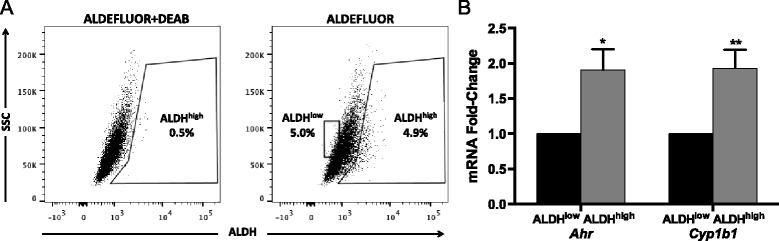
*Ahr* and *Cyp1b1* expression is increased in ALDH1^high^ Hs578T cells. (**a**) ER^−^/PR^−^/HER2^−^ Hs578T cells were stained with ALDEFLUOR™ in the presence or absence of diethylaminobenzaldehyde (DEAB), a specific ALDH inhibitor, and ALDH activity (production of fluorescent substrate) was quantified by flow cytometry. Regions were set using dot plots from DEAB-treated cells. Data are representative of 24 experiments. (**b**) *Ahr* and *Cyp1b1* mRNA expression in sorted ALDH^high^ and ALDH^low^ cells was quantified by RT-qPCR. Data from three independent experiments were analyzed using the Pfaffl method [103], normalized to the *Gapdh* signal, and presented as mean fold-change from ALDH^low^ ± standard error. Asterisks indicate a significant increase in the mRNA fold-change, *P <0.05, **P <0.005

A fluorescence-based ALDH1 enzyme activity assay [19, 20, 23, 52, 53] was used to quantify ALDH1 activity in TNBC Hs578T cells, which express relatively high levels of transcriptionally active AHR [15]. Cells were sorted by flow cytometry into ALDH1^high^ and ALDH1^low^ subsets. Approximately 5 % of Hs578T cells expressed high levels of ALDH1 activity (ALDH^high^; Fig. 1a, right panel), a result consistent with previous studies of BCS_L_Cs [20]. To determine if the *Ahr* and an AHR target gene, *Cyp1b1*, are more highly represented in ALDH^high^ cells, *Ahr* and *Cyp1b1* mRNAs were quantified by RT-qPCR. *Ahr* and *Cyp1b1* mRNAs were significantly higher in ALDH^high^ cells than ALDH^low^ cells (*P* <0.05–0.005; Fig. 1b).

To determine if elevated *Ahr* and *Cyp1b1* expression in ALDH1^high^ cells reflects a role for the AHR in maintaining stem cell properties and if environmental AHR ligands have the potential to increase these properties in TNBCs, AHR expression or activity was modulated with a doxycycline (dox)-inducible *Ahr*-specific shRNA (*shAhr*), AHR inhibitors (CH223191 and CB7993113) [42, 54, 55], or four AHR agonists: (1) 6-formylindolo[3,2-b]carbazole (FICZ), a high affinity AHR ligand, tryptophan photo-metabolite, and potential endogenous ligand [56]; (2) β-naphthoflavone (β-NF), a flavone with moderate affinity for the AHR; (3) 2,3,7,8-tetrachlorodibenzo(*p*)dioxin (TCDD), a high affinity, persistent environmental AHR ligand and ‘gold standard’ AHR ligand; or (4) 7,12 dimethylbenzanthracene (DMBA), a readily metabolizable polycyclic aromatic hydrocarbon.

**Fig. 2:**
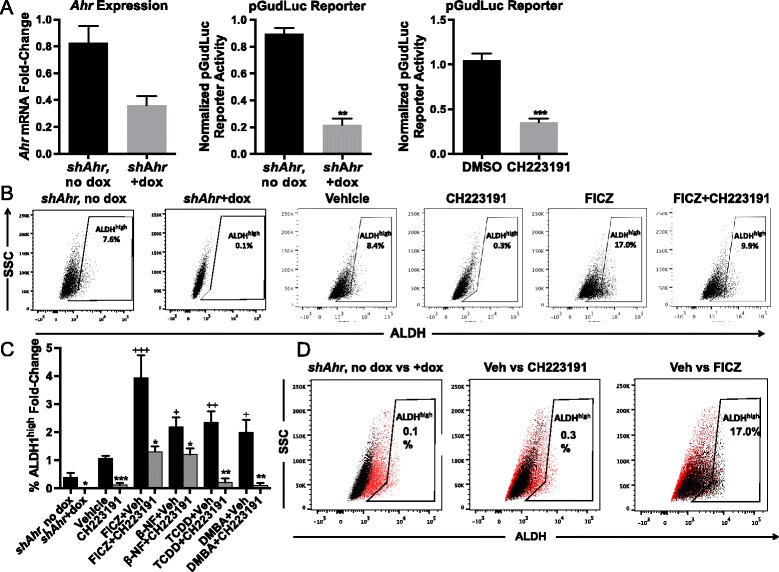
AHR modulation alters ALDH1 activity in Hs578T cells. (**a**) Wildtype or doxycycline (dox)-inducible *shAhr*-transduced Hs578T cells were transfected with *CMV*-*green* control plasmid and AHR-driven *pGudLuc* reporter and treated for 48 hours with 10 μM AHR inhibitor CH223191 or with doxycycline (1.5 μg/mL) to induce the *shAhr. Ahr* mRNA was quantified by RT-qPCR and normalized to *Gapdh* mRNA expression. *pGudLuc* activity was assayed by luminescence and normalized to CMV-green expression. Values were normalized to *Ahr* or *pGudLuc* levels in untreated Hs578T cells. Data are presented as mean ± standard error. Left panel n = 6, middle panel n = 3, right panel n = 6. Asterisks indicate a significant decrease in the mRNA fold-change or reporter activity, *P <0.01, **P <0.001, ***P <0.0001. (**b**) Representative flow cytometry plots of ALDEFLUOR™ staining of wildtype Hs578T cells or dox-inducible *shAhr*-transduced Hs578T cells treated for 48 hours are presented. Dox-inducible *shAhr* transduced Hs578T cells were treated for 48 hours with dox. Hs578T wildtype cells were treated for 48 hours with vehicle, 10 μM CH223191, 1 μM β-NF, 0.5 μM FICZ, 1 nM TCDD, or 1 μM DMBA. Regions representing ALDH^high^ cells were drawn based on the signal generated in the presence of DEAB. (**c**) Hs578T cells were treated as in (b) and assayed for the percentage of ALDH^high^ cells. Data were normalized to results obtained with naïve cells (mean baseline = 4.7 % ALDH^high^ cells) and presented as mean fold-change from naive ± standard error. Number of experiments by condition: shAHR-dox n = 10, shAHR + dox n = 10, DMSO n = 24, CH223191 n = 9, FICZ n = 16, FICZ + CH223191 n = 5, β-NF n = 5, β-NF + CH223191 n = 5, TCDD n = 10, TCDD + CH223191 n = 3, DMBA n = 10, and DMBA + CH223191 n = 4. Asterisks indicate a significant decrease in the percentage of ALDH^high^ cells, *P <0.05, **P <0.001, ***P <0.0005. A cross indicates a significant increase in ALDH^high^ cells, ^+^ *P* <0.05, ^++^ *P* <0.01, ^+++^ *P* <0.005. (**d**) Depicted are flow cytometry dotplots of dox-inducible *shAhr*-transduced Hs578T cells without dox (red dots) versus with dox (black dots), wildtype Hs578T cells treated with vehicle (red dots) versus CH223191 (black dots), or wildtype Hs578T cells treated with vehicle (red dots) versus FICZ (black dots). Data are representative plots of independent experiments of the data presented in (c)

Dox-induced *shAhr* or the AHR-specific inhibitor CH223191 significantly reduced (*P* <0.01–0.0001) *Ahr* expression and AHR-dependent (*pGudLuc*) reporter activity, respectively (Fig. 2a), significantly decreased (*P* <0.05–0.0005) the percentage of ALDH^high^ cells by over 80 % (Fig. 2b, c), and reduced overall ALDH1 activity in the entire Hs578T population (Fig. 2d), suggesting that ‘constitutively active’ (endogenous ligand-activated) AHR maintains baseline ALDH1 levels. Similar data were obtained with our recently described AHR inhibitor, CB7993113 [42] (not shown). Conversely, FICZ, β-NF, TCDD, or DMBA significantly increased the percentage of ALDH1^high^ cells and ALDH1 activity in the entire Hs578T population (*P* <0.05–0.005; Fig. 2b,c, d). In all cases, AHR agonist-induced increases were significantly inhibited by CH223191 (*P* <0.05–0.001). Similar results were obtained with immortalized but non-malignant triple negative MCF-10F cells (Additional file 2: Figure S2) and with ER^+^ luminal-type MCF7 cells [57] (data not shown). These results suggest that AHR ligand-induced ALDH up-regulation is likely generalizable to different breast cancer subtypes.

### Increasing AHR activity increases expression of BCS_L_C-related genes

To determine if several stem cell-associated genes are regulated by the AHR, Hs578T cells were treated for 48 hours with vehicle or FICZ, stained with ALDEFLUOR^TM^, and sorted for ALDH^high^ and ALDH^low^ cells. Consistent with previous studies demonstrating BCS_L_C plasticity [58], pre-sorting Hs578T ALDH^high^ and ALDH^low^ cells prior to treatment and culture for 48 hours was precluded by the tendency for sorted Hs578T subpopulations to revert to the original distribution of ALDH^high^ (~5 %) and ALDH^low^ (~95 %) cells within 24 hours (data not shown).

**Fig. 3:**
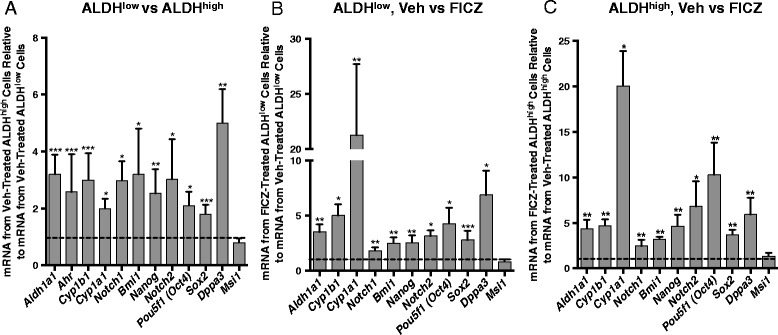
AHR hyper-activation increases expression of stem cell-associated genes. Hs578T cells were treated with vehicle or 0.5 μM FICZ for 48 hours, sorted into ALDH^high^ and ALDH^low^ cell populations, and then assayed by RT-qPCR for the relative levels of the eight stem cell-associated genes indicated. Gene expression was then normalized to *Gapdh* levels and fold-change from vehicle-treated ALDH^low^ or ALDH^high^ cells was calculated. Data from nine independent experiments are presented as the mean fold-change ± standard error for all genes except for *Cyp1a1*, in which six independent experiments are presented. In all cases, statistical significance was determined with the Wilcoxon rank sum test to determine if the distributions of results, relative to 1 as the standard (represented by the dotted line on each graph), are different between the comparison groups. Asterisks indicate a significant increase in the mRNA fold-change, *P <0.05, **P <0.01, ***P <0.005. (**a**) Expression levels of stem cell-associated genes were normalized to expression levels in vehicle-treated ALDH^low^ cells and the distribution of outcomes from vehicle-treated ALDH^high^ versus vehicle-treated ALDH^low^ cells compared. (**b**) Stem cell-associated gene expression levels were normalized to expression levels in vehicle-treated ALDH^low^ cells and the distribution of outcomes from vehicle-treated ALDH^low^ versus FICZ-treated ALDH^low^ cells was compared. (**c**) Stem cell-associated gene expression levels were normalized to expression levels in vehicle-treated ALDH^high^ cells and the distribution of outcomes from vehicle-treated ALDH^high^ versus FICZ-treated ALDH^high^ cells was compared

As expected, vehicle-treated ALDH^high^ cells produced higher levels of *Aldh1a1*, *Ahr*, *Cyp1b1*, and *Cyp1a1* than vehicle-treated ALDH^low^ cells (*P* <0.005; Fig. 3a). *Aldh3a1* mRNA, previously associated with AHR activity [59], was not detected (>33 cycles) in either vehicle or AHR agonist (FICZ or β-naphthoflavone)-treated Hs578T cells (data not shown). ALDH^high^ cells also expressed significantly higher levels of seven of the eight stem cell-associated genes studied (*P* <0.05–0.005) with *Msi1* being the exception (Fig. 3a). As expected from the ALDH enzyme activity assay (Fig. 2), FICZ treatment increased *Aldh1a1*, *Cyp1b1*, and *Cyp1a1* expression in both ALDH^low^ and ALDH^high^ cells (*P* <0.05–0.01; Fig. 3b, c). Consistent with previous studies, AHR ligand induced significantly higher levels of *Cyp1a1* than *Cyp1b1* (*P* <0.05), while baseline *Cyp1b1* levels tended to be higher than *Cyp1a1* levels [15]. FICZ also increased expression of seven of eight stem cell-associated genes in both cell subsets (*P* <0.05–0.005), again with *Msi1* being the outlier (Fig. 3b, c). These results support the hypothesis that constitutively active and/or exogenous agonist-induced AHR up-regulates multiple stem cell-associated genes. Several of these genes express multiple consensus AHR response elements (Table 1), suggesting that they may be directly regulated by the AHR.

**Table 1:** Consensus aryl hydrocarbon receptor response elements (AHREs) in human stem cell- and migration/invasion-associated gene promoters

	Gene	# of Consensus AHREs and location relative to TSS
Standard	*Cyp1b1*	8 (−206, −267, −840, −859, −944, −1028, −1678, −2392)
Stem cell markers	*Aldh1a1*	0
	*Sox2*	7 (−617, −749, −1284, −1430, −1678, −2577, +59)
	*Nanog*	9 (−83, −140, −169, −430, −683, −1076, −2019, −2103, +146)
	*Dppa3*	NA
	*Pou5f1*	3 (−1403, −2203, −2289)
	*Bmi1*	5 (−64, 328, −369, −2275, −2428)
	*Notch1*	13 (−75, −284, −367, −655, −743, −982,-1270, −1838, −1844, −2114, −2140, +52, +196)
	*Notch2*	NA
	*Msi1*	7 (−270, −559, −904, −1821, −2114, −2686, +190)
Invasion and migration markers	*Snai1*	5 (−82, −148, −1836, −1944, +216)
	*Twist1*	NA
	*Vim*	6 (−360, 384, −927, −1003, +224, +294)
	*Twist2*	2 (−314, −2114)
	*Tgfb1*	5 (−836, −1936, −2037, −2190, −2734)
	*Snai2*	5 (−576, −2016, −2821, −2823, +50)
	*Fn1*	1 (−2023)

Consensus AHREs were searched up to 3,000 bp upstream and 300 bp upstream of the transcription start site using Transcriptional Regulatory Element Database (http://rulai.cshl.edu)

**Fig. 4:**
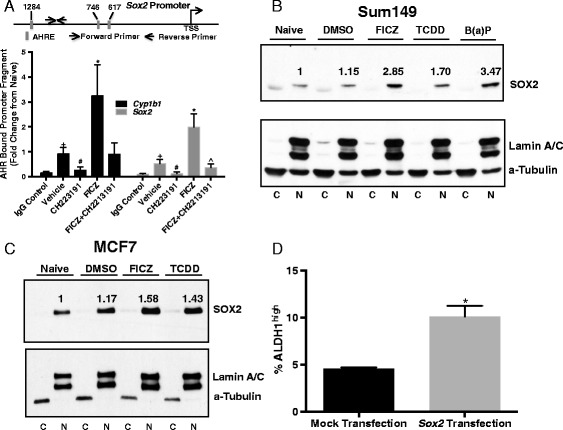
Modulation of AHR activity affects AHR binding to *Cyp1b1* and *Sox2* promoters and SOX2 protein production. (**a**) Hs578T cells were treated with vehicle, 10 μM AHR inhibitor CH223191, 0.5 μM FICZ, or 0.5 μM FICZ + 10 μM AHR inhibitor CH223191 for 48 hours and ChIP assays were performed with human AHR-specific antibody and *Cyp1b1*- or *Sox2*-specific promoters as described in the Materials and Methods. Data are presented as mean fold-change ± standard error, IgG control n = 4, vehicle n = 5, CH223191 n = 4, FICZ n = 5, FICZ + CH223191 = 4. An asterisk indicates a significant increase relative to vehicle controls, *P <0.05. A pound sign indicates a significant decrease relative to vehicle controls, ^#^ *P* <0.05. A cross indicates a significant increase relative to IgG controls, ^+^ *P* <0.005. A caret sign indicates a significant decrease relative to FICZ treatment, ^*P* <0.05. Relative positions of putative AHR response elements and amplified fragments are represented in the embedded map. (**b**) SUM149 or (**c**) MCF-7 cell cytoplasmic and nuclear protein extracts were probed for SOX2 protein expression following treatment with vehicle or AHR agonists: 0.5 μM FICZ, 10 μM B(a)P or 1 nM TCDD. C = cytoplasmic extract, N = nuclear extract. The number above each band indicates fold-change from naïve after normalization to loading control, based on ImageJ densitometry analysis. (**d**) Hs578T cells were transfected with a *CMV* promoter-driven *Sox2* plasmid and ALDH activity assayed 48 hours later. Data from four independent experiments are presented as percent ALDH^high^ ± standard error. Asterisk indicates a significant increase in the %ALDH^high^ cells, *P <0.005

Given the pivotal role for *Sox2* in stem cell self-renewal, BCS_L_C development and breast cancer outcomes [27, 60–63], AHR/*Sox2*-specific ChIP assays were performed to determine if the AHR directly interacts with the *Sox2* promoter. ChIP assays measuring AHR-*Cyp1b1* promoter binding served as positive controls [15]. Indeed, there was a significant basal level of AHR binding to both *Cyp1b1* and *Sox2* promoter fragments (*P* <0.005; Fig. 4a), each of which contains several AHR response elements within 500 bp of the PCR primer binding sites (Table 1). AHR inhibition with CH223191 significantly decreased AHR-*Sox2* and AHR-*Cyp1b1* binding (*P* <0.05). AHR hyper-activation with FICZ significantly increased AHR-*Cyp1b1* and AHR-*Sox2* binding by approximately 3-fold and 2-fold, respectively (*P* <0.05). The AHR-*Sox2* increase was blocked with CH223191 treatment (*P* <0.05; Fig. 4a). Furthermore, treatment of SUM149 cells or MCF-7 cells, which are known to express relatively high SOX2 levels [64], with FICZ, TCDD, or β-NF consistently increased nuclear SOX2 (Fig. 4b, c), a result consistent with increased levels of transcriptionally active SOX2 following AHR hyper-activation. Finally, ectopic *Sox2* expression significantly increased ALDH1 activity (*P* <0.005; Fig. 4d). These data strongly suggest that the AHR directly interacts with *Sox2*, a critical BCS_L_C-associated gene, which in turn regulates ALDH1 expression, an enzyme associated with chemoresistance [53].

### Increasing AHR activity increases expression of migration- and invasion-associated genes

**Fig. 5:**
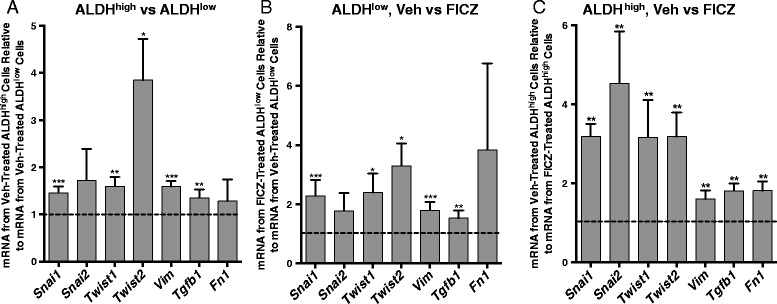
AHR hyper-activation increases expression of migration and invasion-associated genes in Hs578T cells. Hs578T cells were treated with vehicle or 0.5 μM FICZ for 48 hours, sorted into ALDH^high^ and ALDH^low^ cells, and then assayed by RT-qPCR for the relative levels of the seven migration and invasion-associated genes indicated. Gene expression was then normalized to *Gapdh* mRNA levels and fold-change from vehicle-treated ALDH^low^ or ALDH^high^ cells was calculated. Data from nine independent experiments are presented as mean fold-change ± standard error. In all cases, statistical significance was determined with the Wilcoxon rank sum test to determine if the distributions of results, relative to 1 as the standard (represented by the dotted line on each graph), are different between the comparison groups. Asterisks indicate a significant increase in the mRNA fold-change, *P <0.05, **P <0.005, ***P <0.0005. (**a**) Migration- and invasion-associated gene expression levels were normalized to expression levels in vehicle-treated ALDH^low^ cells and the distribution of outcomes from vehicle-treated ALDH^high^ versus vehicle-treated ALDH^low^ cells was compared. (**b**) Gene expression levels were normalized to expression levels in vehicle-treated ALDH^low^ cells and the distribution of outcomes from vehicle-treated ALDH^low^ versus FICZ-treated ALDH^low^ cells was compared. (**c**) Gene expression levels were normalized to expression levels in vehicle-treated ALDH^high^ cells and the distribution of outcomes from vehicle-treated ALDH^high^ versus FICZ-treated ALDH^high^ cells was compared

BCS_L_Cs are more invasive than the bulk tumor population and have increased expression of migration- and invasion-associated markers [21, 22, 28, 29, 53, 58, 65]. To determine if the increase in stem cell markers described above correlates with markers of migration and invasion, Hs578T cells were treated with vehicle or FICZ for 48 hours, sorted for ALDH^high^ and ALDH^low^ cells, and evaluated for expression of seven genes associated with increased tumor migration and/or invasion. As seen for stem cell markers (Fig. 3), ALDH^high^ cells expressed significantly higher levels (*P* <0.005–0.0005) of *Snai1*, *Twist 1*, *Twist2*, *Tgfb1*, and *Vim* than ALDH^low^ cells, with *Twist2* showing the greatest fold-change (Fig. 5a). Although *Snai2* and *Fn1* tended to be higher in ALDH^high^ cells, neither was statistically significant in nine independent experiments. These data are consistent with the BCS_L_C properties of ALDH^high^ cells. AHR hyper-activation with FICZ significantly (*P* <0.05–0.0005) increased *Snai1*, *Twist1*, *Twist2*, and *Vim* in both ALDH^high^ and ALDH^low^ cells (Fig. 5b,c) and *Tgfb1* was marginally increased in FICZ-treated ALDH^low^ cells (Fig. 5c).

As a functional readout of migration, the effects of AHR modulation on the ability of SUM149 cells to migrate in a 48 hour scratch-wound assay were determined. SUM149 cells were chosen for this experiment since, unlike Hs578T cells, ALDH^high^ SUM149 cells remained ALDH^high^ in vitro for at least 96 hours, unless the AHR inhibitor, CH223191 was added (Additional file 3: Figure S3A). ALDH^low^, SUM149 cells tended to revert to ALDH^high^ phenotype but this reversion was inhibited by CH223191 treatment (Additional file 3: Figure S3B). Similar results describing the plasticity of stem-like cells have been previously reported [58].

**Fig. 6:**
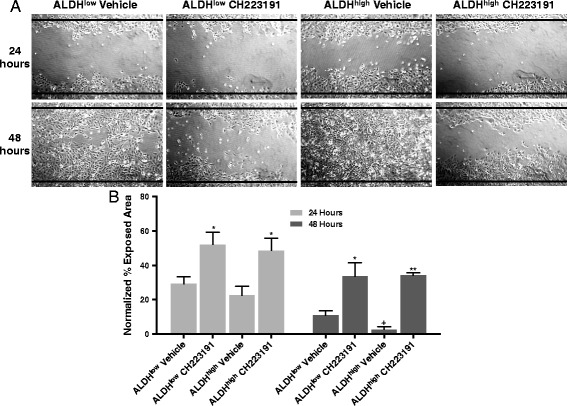
AHR down-regulation decreases migration of SUM149 cells. (**a**) Presented are representative images of SUM149 cell migration at 24 and 48 hours after cells were sorted into ALDH^high^ and ALDH^low^ populations, cultured to confluence, scratched, and treated with vehicle or 10 μM CH223191. Data are representative of three independent experiments. Black lines indicate the borders of the original scratch. (**b**) SUM149 cells were treated as in (a) and percent exposed area was quantified. Data from three experiments were normalized to results obtained with naïve cells and presented as mean percent exposed area ± standard error. Asterisks indicate a significant increase in exposed area, *P <0.05, **P <0.0005. A cross indicates a significant decrease in exposed area, ^+^ *P* <0.05

As expected, vehicle-treated ALDH^high^ cells ‘repaired’ the wound significantly faster than vehicle-treated ALDH^low^ cells, as quantified by a decrease in exposed surface area (Fig. 6; *P* <0.05 at 48 hours). Furthermore, wound repair with both subpopulations was significantly inhibited by CH223191 treatment (Fig. 6; *P* <0.05–0.0005). Similar data were obtained with unsorted Hs578T and SUM149 cells and with another AHR inhibitor, CB7993113 (not shown). No cell divisions were observed over this 48-hour period as assessed by CFSE staining and analysis by flow cytometry (not shown). In addition, both 0.5 μM FICZ and 1 nM TCDD significantly accelerated migration of unsorted, ALDH^low^ (not shown), and ALDH^high^ SUM149 subsets (Additional file 4: Figure S4). A significant increase in migration rate also was seen for ALDH^high^ cells following a 48-hour treatment with a lower TCDD dose (0.2 nM, not shown).

### Generalization of the correlation between *Ahr* or *Cyp1b1* and BCS_L_C- and invasion/migration-associated genes

The experiments described above confirm that AHR hyper-activation with FICZ induces both BCS_L_C- and migration/invasion-associated genes in an AHR-dependent fashion in Hs578T cells. If these associations are generalizable to other breast cancer cell lines, then it would be predicted that *Ahr* expression and expression of *Cyp1b1*, as a marker for AHR activity, would correlate, in multiple breast cancer cell lines, with expression of the BCS_L_C- and migration/invasion-associated gene sets identified in Hs578T cells. For such an analysis, we used microarray/RNA-seq data compiled by the Broad Institute on 79 primary human breast cancer cell lines, i.e. the Cancer Cell Line Encyclopedia (CCLE) [66]. Use of *Cyp1b1* as a marker for AHR activity in this context is supported by (1) our findings [15], and those of others [67], demonstrating that baseline *Cyp1b1* mRNA levels are maintained in part by ‘constitutively active’ AHR in human breast cancer cell lines, and (2) the observation that, of all breast cancer cell lines in the CCLE, the nearest neighbor to *Ahr* of >20,000 gene probes is *Cyp1b1* (*P* = 0.0019; this is not to say that there are no other factors regulating *Cyp1b1* expression [67]). Gene set enrichment analyses (GSEA) were performed with the aim of testing whether the gene set listed in Table 1 is significantly and coordinately correlated with *Ahr* or *Cyp1b1* expression. Indeed, *Ahr* expression was significantly correlated (false discovery rate = 0.025) with the putative AHR target gene set shown in Table 1 (Additional file 5: Figure S5A). Similarly, there was a significant correlation between *Cyp1b1* and the expression of the putative AHR target gene set (FDR = 0.021; Additional file 5: Figure S5B). Interestingly, the ‘outlier’ with a negative correlation score for both the *Ahr* and *Cyp1b1* analyses, was *Msi1* (Additional file 5: Figure S5A and S5B, red arrow), the one stem cell-associated gene we tested that did not increase following AHR hyper-activation (Fig. 3).

To generalize results to primary human cancers, a similar GSEA analysis was performed using transcriptomic data from 977 primary human breast cancers catalogued in the Cancer Genome Atlas (TCGA) database [68] and 995 primary human breast cancers in the Curtis database [69]. As shown for cell lines in the CCLE, there was a significant association (FDR = 0.047) between *Ahr* expression and the gene set listed in Table 1 (Additional file 6: Figure S6A). A stronger association (FDR = 0.0001) was seen between *Cyp1b1* expression and expression of the putative AHR target gene set (Additional file 6: Figure S6B). As with the CCLE database, *Msi1* was not correlated with either *Ahr* or *Cyp1b1* in the TCGA database (Red arrows, Additional file 6: Figure S6A, S6B). Similar data were obtained using the Curtis dataset (not shown). Collectively, data mined from three large breast cancer databases (CCLE, TCGA, and Curtis) show a significant and generalizable association between *Ahr* or AHR activity (*Cyp1b1* expression) and cancer stem cell- and migration/invasion-associated gene sets, an outcome consistent with regulation of these genes by a constitutively active (i.e. endogenous AHR ligand-activated) AHR.

### Decreasing AHR activity decreases tumorsphere formation

**Fig. 7:**
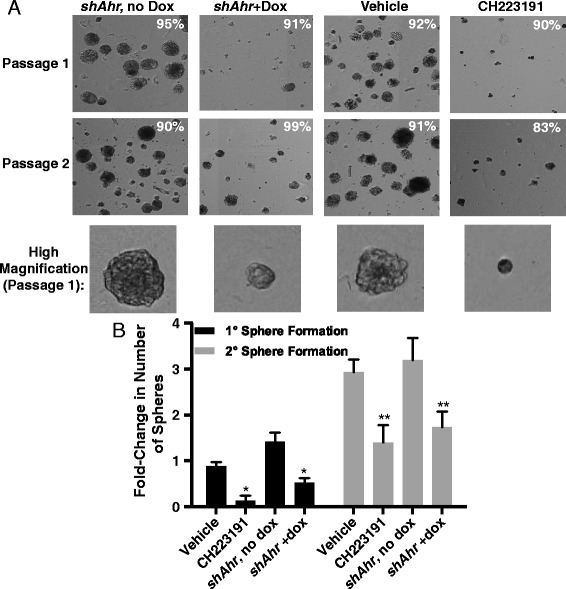
AHR down-regulation decreases Hs578T tumorsphere formation. (**a**) Dox-inducible *shAhr*-transduced Hs578T (‘*shAhr*’) or wildtype Hs578T cells were left untreated or treated for 48 hours with vehicle, doxycycline, or 10 μM CH223191 as indicated and cultured in Mammocult media under ultra-low adherence conditions. Representative images of primary (day 8) and secondary (day 16, following passage at day 8) tumorspheres are presented. The percentage of viable cells is included on each image. Vehicle and CH223191 treatment groups are representative of six independent experiments. *shAHR*, no dox and *shAHR* + dox treatment groups are representative of five independent experiments. (**b**) Hs578T cells were treated as in (a) and tumorsphere formation was quantified. Data from six experiments were normalized to results obtained with naïve cells and presented as mean fold-changes from naive ± standard error. Asterisks indicate a significant decrease in the percentage of tumorspheres, *P <0.05, **P <0.005

BCS_L_C can form tumorspheres and produce progenitor cells in ultra-low adherence conditions over several passages [20, 31, 70–73]. To determine if the AHR contributes to this functional readout of BCS_L_Cs, Hs578T cells were cultured in Mammocult media under ultra-low adherence conditions and AHR activity and expression were modulated with CH223191 or with a dox-inducible *shAhr*. Both the size and total number of tumorspheres were significantly reduced (*P* <0.05–0.005) by CH223191 or a dox-induced *shAhr* in primary, secondary (Fig. 7a,b), tertiary, and quaternary (not shown) cultures. No effect on cell viability (trypan blue exclusion) was seen (the percent viability is indicated in the upper right corner of each image in Fig. 7a). Similar results were obtained with CB7993113 (not shown). These results suggest that the AHR regulates tumorsphere formation and the ability of BCS_L_Cs to (asymmetrically) divide and/or differentiate into progenitor cells in low-adherence, selective conditions, and/or controls the ability of progenitor cells to divide.

### AHR controls expression of cancer stem cell-associated properties in an inflammatory breast cancer cell line

**Fig. 8:**
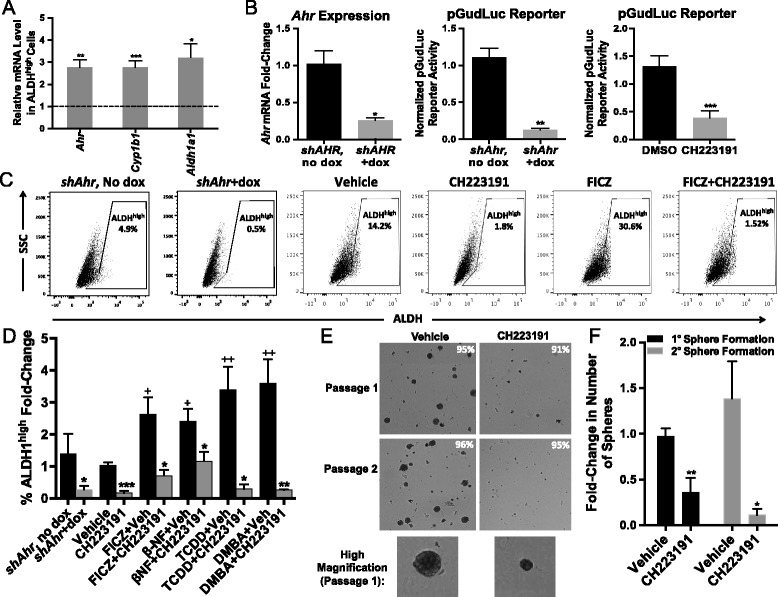
AHR modulation affects markers associated with BCS_L_C in SUM149 cells. (**a**) *Ahr*, *Cyp1b1* and *Aldh1a1* mRNA expression in sorted ALDH^high^ and ALDH^low^ SUM149 cells was quantified by RT-qPCR. Data from three independent experiments were analyzed using the Pfaffl method, normalized to the *Gapdh* signal, and presented as mean fold-change from ALDH^low^ ± standard error. Asterisks indicate a significant increase in the mRNA fold-change, *P <0.05, **P <0.01, ***P <0.005. (**b**) Wildtype or dox-inducible *shAhr*-transduced SUM149 cells were transfected, treated and quantified as indicated in Fig. 2a. Data are presented as mean ± standard error. Left panel n = 5, center panel n = 4, right panel n = 6. Asterisks indicate a significant decrease in the mRNA fold-change or reporter activity, *P <0.01, **P <0.001, ***P <0.0001. (**c**) Representative flow cytometry plots of ALDEFLUOR™ staining of dox-inducible *shAhr*-transduced SUM149 cells cultured, treated, and depicted as described in Fig. 2b. (**d**) Dox-inducible *shAhr*-transduced or wildtype SUM149 cells were treated, stained, and quantified as in Fig. 2c. Data were normalized to results obtained with naïve cells (mean baseline =10.4 % ALDH^high^ cells) and presented as mean fold-change from naive ± standard error. shAHR-dox n = 4, shAHR + dox n = 4, DMSO n = 12, CH223191 n = 5, FICZ n = 6, FICZ + CH223191 n = 5, β-NF n = 4, β-NF + CH223191 n = 4, TCDD n = 8, TCDD + CH223191 n = 3, DMBA n = 5, and DMBA + CH223191 n = 3. Asterisks indicate a significant decrease in the percentage of ALDH^high^ cells, *P <0.05, **P <0.01, ***P <0.0005. A cross indicates a significant increase in ALDH^high^ cells, ^+^ *P* <0.01, ^++^ *P* <0.005. (**e**) Representative images of primary (day 8) and secondary (day 16) tumorspheres after SUM149 cells were treated for 48 hours with vehicle or 10 μM CH223191. The percent viable cells are included on each image. Data are representative of four independent experiments. (**f**) SUM149 cells were treated as in (e) and tumorsphere formation was quantified. Data from four experiments were normalized to results obtained with naïve cells and presented as mean fold-change from naive ± standard error. Asterisks indicate a significant decrease in the percentage of tumorspheres, *P <0.05, **P <0.01

IBC is a particularly aggressive form of cancer characterized by a ~50 % survival rate at 2 years [74]. To determine if AHR control of stem cell characteristics is generalizable to this cancer subtype, SUM149 cells, derived from an IBC, were studied for expression of *Ahr* and *Cyp1b1* in ALDH^high^ and ALDH^low^ subpopulations, for the contribution of the AHR to ALDH1 activity, and for the ability to form tumorspheres. As shown for TNBC Hs578T cells, ALDH^high^ SUM149 cells expressed significantly higher *Ahr* and *Cyp1b1* levels than ALDH^low^ cells (*P* <0.05–0.005; Fig. 8a). CH223191 or *Ahr*-specific shRNA significantly decreased AHR activity or expression greater than 60 % (*P* <0.01–0.0001; Fig. 8b), the percentage of ALDH^high^ cells by over 80 % (*P* <0.05–0.0005; Fig. 8c,d), and overall ALDH1 activity in the entire population (not shown). Conversely, FICZ, β-NF, TCDD, and DMBA significantly increased the percentage of ALDH^high^ cells (*P* <0.01–0.005; Fig. 8c,d) and CH223191 treatment in tandem significantly reduced this increase (*P* <0.05–0.01; Fig. 8c,d). Finally, fewer and smaller tumorspheres were formed following CH223191 treatment (*P* <0.05–0.01; Fig. 8e,f). No changes in cell viability were detected (the percent viability is indicated in the upper right corner of each image in Fig. 8e). These data parallel those found with Hs578T cells (Fig. 7) and suggest that AHR control of these stem cell properties is generalizable to other ER^−^ breast cancer subtypes.

### Decreasing AHR activity decreases chemoresistance, a hallmark of BCS_L_Cs

**Fig. 9:**
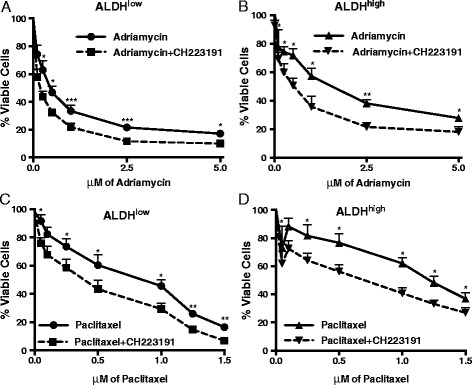
AHR down-regulation decreases chemotherapeutic resistance of both ALDH^high^ and ALDH^low^ Hs578T cells. MTT assays were used to measure cell viability after Hs578T cells were sorted into ALDH^low^ (**a** and **c**) and ALDH^high^ (**b** and **d**) populations and treated with adriamycin (**a** and **b**) or paclitaxel (**c** and **d**) with and without 10 μM CH223191 for 24 hours. CH223191 treatment alone did not affect cell viability (≥95 % viability in the presence or absence of CH223191 only). Data from six independent experiments were normalized to vehicle-treated cells and presented as mean percent viable cells ± standard error. Asterisks indicate a significant increase in cell death, *P <0.05, **P <0.005, ***P <0.005

Chemoresistance is another widely studied functional BCS_L_C marker [21, 22, 53, 72, 73, 75, 76]. To determine if the AHR influences chemoresistance, ALDH^high^ and ALDH^low^ Hs578T cells were treated with titrated doses of adriamycin or paclitaxel, chemotherapeutics with distinct mechanisms of action, with or without CH223191. Cell viability was assayed 24 hours later. As expected of BCS_L_Cs, ALDH^high^ cells were more resistant to the chemotherapeutics than ALDH^low^ cells (see half maximal effective concentrations (EC_50_) in Table 2). CH223191 had no effect on viability (not shown). However, CH223191 significantly (*P* <0.05–0.005) increased sensitivity to both adriamycin and paclitaxel in both ALDH^low^ and ALDH^high^ cells (Fig. 9). The EC_50_ of adriamycin-treated ALDH^high^ cells (EC_50_ = 1.99 μM) was three times greater than that of adriamycin + CH223191-treated cells (EC_50_ = 0.60 μM; Fig. 9b; Table 2). These results are consistent with previous reports demonstrating AHR control of chemotherapeutic-induced breast cancer cell apoptosis [77]. Furthermore, they indicate that migration/invasion-associated genes, and functional markers of BCS_L_Cs (tumorsphere formation, rapid migration, chemoresistance) are influenced by the AHR.

**Table 2:** Half maximal effective concentrations (EC_50_) of two chemotherapeutics in the presence or absence of an AHR inhibitor

Cell population	Drug treatment	Chemotherapeutic EC_50_ (μM)
ALDH^low^	Paclitaxel	0.81
ALDH^low^	Paclitaxel + CH	0.56
ALDH^high^	Paclitaxel	1.21
ALDH^high^	Paclitaxel + CH	0.76
ALDH^low^	Adriamycin	0.55
ALDH^low^	Adriamycin + CH	0.20
ALDH^high^	Adriamycin	1.99
ALDH^high^	Adriamycin + CH	0.60

EC_50_ values were calculated from the data presented in Fig. 9

### *shAhr*-mediated AHR knockdown decreases expansion of tumors initiated with ALDH^high^ and ALDH^low^ SUM149 cells

**Fig. 10:**
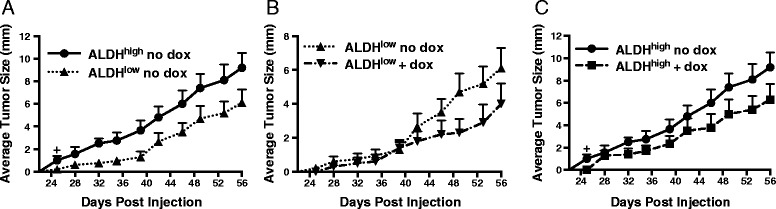
AHR down-regulation decreases tumor formation in xenograft mice. (**a**) Doxycycline-inducible *shAhr-*expressing SUM149 cells were sorted for ALDH^high^ or ALDH^low^ activity and 3,000 cells were grafted into the mammary fat pads of NOD/SCID mice (10 mice/group). Tumor volumes were measured over the next 56 days. Data are presented as mean tumor volume ± standard error; *P* <0.0001. The average rate of tumor growth of tumors initiated with ALDH^high^ cells (0.26 mm/day) was significantly different than the rate of growth of tumors initiated with ALDH^low^ cells (0.19 mm/day), *P* <0.0005. A cross indicates a significant increase in average tumor size beginning at day 25, ^+^ *P* <0.01. (**b**) Doxycycline-inducible *shAhr-*expressing SUM149 cells were sorted for low ALDH expression and 3,000 cells were grafted into the mammary fat pads of NOD/SCID mice. Mice then were given water + 5 % sucrose or water with doxycycline + 5 % sucrose and tumor volumes were quantified over the next 56 day period. The average rate of tumor growth of ALDH^low^ cells in control mice (0.19 mm/day) was significantly different than that of ALDH^low^ cells in dox-treated mice (0.11 mm/day), *P* <0.0005. (**c**) Doxycycline-inducible *shAhr-*expressing SUM149 cells were sorted for high ALDH expression and 3,000 cells were grafted into the mammary fat pads of NOD/SCID mice. Mice were then given water + 5 % sucrose or water with doxycycline + 5 % sucrose. Tumor volumes were measured over the next 56 day period. The average rate of tumor growth in water-treated control mice (0.26 mm/day) was significantly different than the rate of tumor growth in dox-treated mice (0.18 mm/day), *P* <0.0005. A cross indicates a significant increase in tumor size beginning at day 25, ^+^ *P* <0.01

**Fig. 11:**
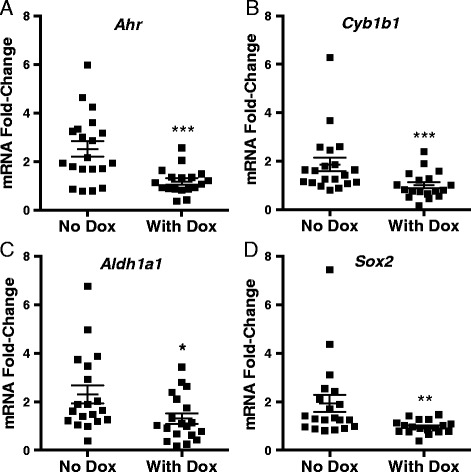
AHR down-regulation decreases expression of *Ahr*, *Aldh1a1*, *Cyp1b1*, and *Sox2* in xenografted mouse tumors. Twenty NOD/SCID mice were injected with 3,000 dox-inducible *shAhr*-transduced ALDH^high^ or ALDH^low^ SUM149 cells as described in Fig. 10. Half of the mice were provided with water containing 2 mg/mL doxycycline to induce the *shAhr* and tumors were harvested after 42–72 days when they reached 15 mm in total size (control mice n = 20, mice with doxycycline treatment n = 19). (**a**) *Ahr*, (**b**) *Cyp1b1*, (**c**) *Aldh1a1*, and (**d**) *Sox2* mRNA expression levels were assayed by RT-qPCR. Since the AHR controls expression of *Cyp1b1*, *Aldh1a1*, and *Sox2* in both ALDH^high^ and ALDH^low^ cells (e.g. Fig. 3), data generated with tumors from ALDH^high^ and ALDH^low^ tumors were pooled for statistical purposes. Data are presented as fold-change relative to the average CT value from control mice, i.e. no dox, grafted with ALDH^low^ cells ± standard error. Asterisks indicate a significant decrease in the mRNA fold-change, *P <0.05, **P <0.01, ***P <0.005

Cancer stem cells tend to generate tumors more efficiently in vivo than non-cancer stem cells [18–20, 31, 72]. To determine if AHR, which increases expression of stem cell-associated properties in vitro, influences tumor cell fate in vivo, SUM149 cells, stably transduced with a dox-inducible *shAhr* (Fig. 8), were sorted and 3,000 ALDH^high^ and ALDH^low^ cells were injected into the right and left mammary fat pads, respectively, of female NOD/SCID mice. Half of the mice were given doxycycline-containing water to induce the *shAhr*. ALDH^high^ cells generated palpable tumors more rapidly and these tumors grew faster than ALDH^low^ cells (growth rates of 0.26 vs. 0.19 mm/day, *P* <0.0001; Fig. 10a). Furthermore, dox-induced *shAhr* significantly reduced growth rates from 0.26 to 0.18 and from 0.19 to 0.11 mm/day in ALDH^high^ and ALDH^low^ cells, respectively (*P* <0.0005; Fig. 10b,c). Consistent with in vitro experiments, *Ahr*, *Cyp1b1*, *Aldh1a1*, and *Sox2* mRNA levels were reduced in tumors from doxycycline-treated mice (Fig. 11).

**Fig. 12:**
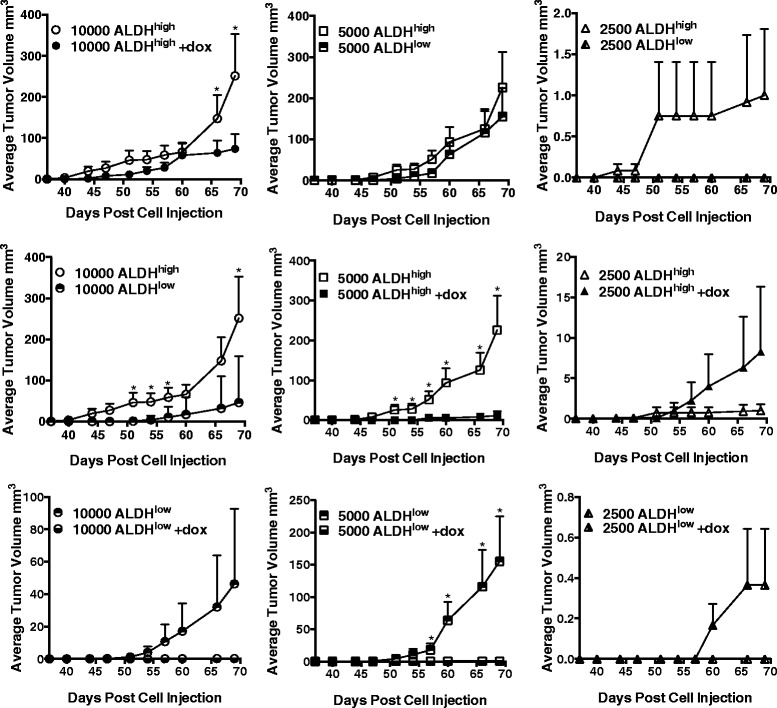
AHR down-regulation decreases the efficiency of tumor formation in vivo. Titered numbers (10,000, 5,000, or 2,500) of sorted ALDH^high^ or ALDH^low^ dox-inducible *shAhr*-transduced SUM149 cells were grafted into the mammary fat pads of NOD/SCID mice (six mice/group). Mice were given water + 5 % sucrose or water with doxycycline + 5 % sucrose to induce the *shAhr*. Data are presented as mean tumor volume ± standard error. Asterisks indicate a significant difference in tumor volume, *P* <0.05. Statistical analyses were not performed when none of the mice in a given control group developed tumors at that time point

**Fig. 13:**
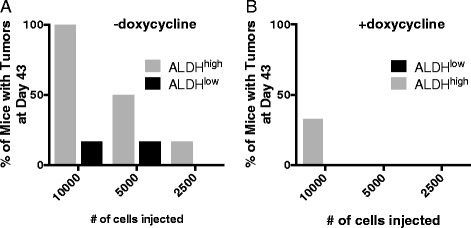
Percent of mice bearing tumors 43 days after receiving orthotopic xenografts of 10,000, 5,000, or 2,500 ALDH^high^ or ALDH^low^ SUM149 cells. The percentage of mice with palpable tumors at day 43 of the experiment presented in Fig. 12 was determined as a measure of tumor initiation efficiency. Mice were given either (**a**) water + 5 % sucrose (n = 20) or (**b**) water with doxycycline + 5 % sucrose to induce expression of *shAHR* (n = 20)

Although ALDH1 activity and *Aldh1* expression have been identified as a valid marker for BCS_L_Cs [19, 23, 50, 53, 78] and despite ALDH^high^ cells having formed tumors sooner than ALDH^low^ cells in the experiment described above, we wanted to confirm that ALDH^high^ cells, in our hands, exhibit a hallmark property of cancer stem cells in vivo, i.e. efficient formation of tumors following xenografting. Therefore, SUM149 cells, stably transduced with a different doxycycline-inducible *shAHR*, were sorted into ALDH^high^ and ALDH^low^ subpopulations and xenografted at titered numbers (10,000, 5,000, 2,500 cells) into the mammary fat pads of female NOD/SCID recipients (six mice/group). Tumor volume was then tracked over a 69-day period. As predicted of tumors derived from cells with cancer stem-like properties, tumors generated from ALDH^high^ cells were detected sooner than tumors generated from ALDH^low^ cells at each respective cell number, thereby demonstrating a consistently higher efficiency of tumor initiation (Figs. 12 and 13). Tumors grew faster after xenograft of 10,000 ALDH^high^ as compared with ALDH^low^ cells. Furthermore, induction of *shAhr* with doxycycline significantly delayed tumor formation and subsequent growth of tumors generated from both ALDH^high^ cells and ALDH^low^ cells regardless of cell number transferred to recipients. These data strongly support the use of ALDH activity as a marker for breast cancer cells with cancer stem cell-like properties and the conclusion that AHR influences the efficiency with which all cells along a continuum of low to high ALDH expression can initiate tumors.

## Discussion

Accumulating data suggest that the AHR plays an important role in breast cancer, in general, and in progression to end-stage invasion and migration in particular. For example, the AHR is hyper-expressed and transcriptionally active in most TNBC and IBC cell lines, and its expression is associated with tumor invasion [10, 13, 15, 79]. The data presented here strongly suggest that the AHR drives tumorigenesis in part through induction or maintenance of cells with cancer stem cell-like properties.

The involvement of the AHR in BCS_L_C biology is suggested by its emerging role in normal tissue-specific stem cell development. For example, the AHR, presumably activated by endogenous ligand(s), helps maintain hematopoietic stem cell self-renewal and block differentiation [36, 37, 80], and drives bipotential blood stem cell differentiation [39]. AHR repression in embryonic stem cells likely maintains pluripotency, and the AHR controls embryonic stem cell differentiation into cardiomyocytes [38]. Data presented here extend these studies by demonstrating that the AHR is involved in the phenotype (ALDH1 activity and *Aldh1a1* expression), genomics (up-regulation of stem cell- and migration/invasion-associated genes), and function (migration, chemoresistance, tumorigenicity) of BCS_L_Cs.

Elevated ALDH expression identifies BCS_L_Cs and is associated with increased expression of chemoresistance proteins, increased tumor cell invasion, higher tumor grade, and poor survival in breast cancer patients [19, 21, 28, 32, 53]. Indeed, in our hands, ALDH^high^ cells exhibited increased chemoresistance (Fig. 9), elevated expression of stem cell- and migration/invasion-associated genes (Figs. 3 and 5), faster migration (Fig. 6), higher tumor-initiating capacity and increased tumor growth rates in vivo (Figs. 10, 12 and 13). AHR control of chemoresistance is particularly interesting given important recent studies demonstrating that TCDD decreases and AHR inhibition increases apoptosis induced by UV light or chemotherapeutics in six breast cancer cell lines [77]. AHR-mediated chemoresistance takes on even greater significance given recent studies showing that chemoresistance may be a more meaningful marker of metastatic behavior than markers of epithelial-to-mesenchymal transition [25]. These data support the hypothesis that AHR inhibitors may represent effective, targeted therapeutics when used in combination with conventional chemotherapeutics. Collectively, these data strongly support the conclusion that ALDH^high^ cells are at least breast cancer stem-like cells if not bona fide breast cancer stem cells.

We previously demonstrated that constitutively active AHR in breast cancer lines preferentially drives *Cyp1b1* expression while an exogenous ligand, e.g. DMBA, tends to induce greater fold-increases in *Cyp1a1* than *Cyp1b1* [15]. Interestingly, higher relative levels of *Cyp1b1* were noted in ALDH^high^ cells, as compared with ALDH^low^ cells (Fig. 3a), suggesting the possibility that a higher level of AHR activity, as represented by baseline *Cyp1b1* levels, characterizes ALDH^high^-stem like cells.

AHR hyper-activation with FICZ increased expression of *Aldh1a1* and stem cell-associated genes demonstrating a causal relationship between AHR activity and expression of these genes (Fig. 3b,c). This gene set has been implicated in generating both normal tissue stem cells and BCS_L_Cs. *Notch1* and *Notch2* are critical to symmetric and asymmetric cell division, stem cell differentiation in embryonic and adult stem cells [26, 81], and are potential therapeutic targets [76, 82]. *Bmi1* is required for the maintenance of somatic stem cells through repression of cellular senescence and cell death [83], and is involved in the control of BCS_L_C growth, chemoresistance, and tumorsphere formation [84]. *Stella* is involved in maintenance of gene-specific DNA methylation in the early embryo, and is a marker for some BCS_L_C types [85]. *Sox2*, *Oct4* and *Nanog* are traditional embryonic stem cell markers used to reprogram cells to a pluripotent state, and are expressed at elevated levels in BCS_L_Cs [27, 30, 85, 86]. *Sox2* is up-regulated in TNBCs and has been implicated in tumorsphere formation and control of tumor initiation [27, 62, 63]. Therefore, the finding that the AHR directly interacts with the *Sox2* promoter (Fig. 4a) strongly suggests that the AHR is at the apex of an important signaling pathway that controls cancer progression by increasing phenotypic and functional expression of cancer stem cell-associated markers within the tumor cell population.

Our data also suggest that the AHR plays a key role in regulating BCS_L_C migration. *Snail*, *Slug*, *Twist1*, *Twist2*, *Tgfb1*, and *Fibronectin* (*Fn1*), which contribute to cell invasion and cell migration [28, 87, 88], are all up-regulated in FICZ-treated ALDH^high^ cells (Fig. 5c). As would be predicted from these results, AHR inhibition slows (Fig. 6) and AHR hyper-activation accelerates (Additional file 4: Figure S4) cell migration in the scratch-wound assay.

Importantly, these findings on AHR-regulated genes appear generalizable since strong correlations were seen between *Ahr* or *Cyp1b1* and the stem cell- and migration/invasion-associated gene sets in databases of 79 human breast cancer cell lines characterized in the CCLE and over 1850 primary human breast cancers catalogued in the TCGA and Curtis databases (Additional files 5 and 6: Figure S5 and S6). Furthermore, these results suggest the possibility that the AHR contributes to cell invasion and migration through up-regulation of stem cell- and invasion/migration-associated genes.

Consistent up-regulation of CYP1B1 in breast cancers [89] suggests that this enzyme plays an important role in cancer, potentially by influencing cell migration [90]. It is, therefore, formally possible that at least some of the effects observed here reflect AHR ligand binding to CYP1B1. While this possibility cannot be ruled out, particularly for ligands such as FICZ, which are metabolized by CYP1B1 [91], it seems unlikely as a general rule since TCDD, which is not metabolized by CYP1B1 and does not bind CYP1B1 (data not shown), generates the same outcomes (increase in stem cell-associated genes, cell migration) as the other ligands.

AHR inhibition or knockdown in either Hs578T or SUM149 cells significantly reduced the number and size of tumorspheres formed in low adherence conditions over several generations (Figs. 7 and 8e,f). The formation of these colonies is generally considered to be a function of asymmetric BCS_L_C division and production of progenitor cells which constitute the majority of the cells in the spheres [31, 70, 72]. Therefore, it is possible that the AHR controls the asymmetric differentiation of BCS_L_C and/or the growth of their progenitors.

Furthermore, AHR knockdown with either of two *shAhr* constructs significantly slowed the initiation and outgrowth of both ALDH^low^ and ALDH^high^ cell-derived tumors (Figs. 10b, c, 12b, c, 13). This decrease in tumor outgrowth was accompanied by a decrease in *Cyp1b1*, *Aldh1a1*, and *Sox2* expression (Fig. 11), further linking AHR activity to expression of these genes.

Results presented here are reminiscent of several studies demonstrating that baseline (endogenous ligand-induced) AHR activity in immortalized cells favors tumor growth or aggressive behavior [12–14, 92–97]. Paradoxically, several studies indicate that exogenous AHR ligands can reduce tumor growth or invasion [41, 92, 96, 97]. As elegantly described in a recent review [98], these seemingly contradictory results may, in part, reflect context- or tumor stage-specific differences. For example, AHR agonists may inhibit growth in ER^+^ breast cancers in part through AHR-mediated down-regulation of ER expression or activity [41]. However, in similar cell types [47, 99, 100], similar AHR agonist- and antagonist-mediated outcomes could be due to more subtle effects on AHR activation or signaling. For instance, it has been postulated that, while endogenous AHR ligands drive signaling towards, for example, increased invasion, exogenous AHR ligands ‘divert’ [10] or ‘disrupt’ [92] the response towards signaling pathways which oppose tumor invasion, e.g. differentiation [47]. Furthermore, exogenous ligands, e.g. Tranilast, that decrease invasion [99, 100], may act as partial agonists that compete with endogenous ligands for AHR binding but which are weaker activators of AHR transcriptional activity, thereby reducing baseline AHR signaling [101]. Finally, outcomes may be ligand-, cell subset-, or dose-specific. Thus, high affinity AHR ligands, such as TCDD, induce stem cell characteristics including ALDH expression and accelerated migration, particularly at low doses (e.g. 0.2–1 nM; Additional file 4: Figure S4, and data not shown), while higher doses (10 nM) may reduce invasiveness of the majority non-BCS_L_C population [47].

Finally, a limited number of previous studies have addressed the role of the AHR in breast cancer stem cell generation [96, 97, 99, 102]. While these studies all point towards a role for the AHR in cancer stem-like cell generation, there is as yet no clear consensus on how this occurs or even on whether the AHR favors or inhibits BCS_L_C production/function. For example, Zhao et al. [96] showed that AHR activation with β-NF or 3-MC or over-expression of a PasB mutant AHR decreased tumorsphere formation; in our hands, only 3-MC reduced secondary tumorsphere formation in SUM149 and MCF-7 cells (data not shown). In what may seem like a contradiction, Zhao et al. [97] later published that MCF-7 mammosphere formation was suppressed by AHR inhibition with CH223191 as well as by siRNA-mediated AHR knockdown in MDA-MB-453 cells. In Dubrovska et al. [102], AHR inhibitors reduced the percentage of ALDH^high^ MCF-7 cells in tamoxifen-resistant MCF-7 (as shown in our studies with triple negative Hs578T and SUM149 cells) but produced the opposite effect in wildtype MCF-7 cells. At least some of these differences can be attributed to the different subtypes of breast cancer cells (i.e. ER^+^, Luminal A-type or Her-2 over-expressing MCF-7 cells versus ER^−^, basal-like, triple negative Hs578T and SUM149 cells). In any case, further experimentation is required to determine how the AHR influences ‘stem-ness’ in breast cancer cells.

## Conclusions

Studies presented here indicate that the AHR influences, in TNBC and IBC cells, critical markers associated with ‘stem-ness’. The ability of several exogenous AHR ligands, including TCDD and DMBA to up-regulate phenotypic, genomic, and/or functional markers of BCS_L_Cs strongly suggests the potential for ubiquitous environmental AHR ligands to accelerate progression to lethal, invasive cancers. Furthermore, the demonstration that AHR inhibition significantly reduces expression of these phenotypic and functional cancer stem cell markers encourages the testing of AHR inhibitors, for example, to significantly increase the sensitivity of BCS_L_Cs to conventional chemotherapeutics. In general, these results suggest that non-toxic AHR modulators may represent important therapeutics for otherwise refractory TNBC and IBC, and potentially for brain and other cancers in which the AHR appears to play a role.

## Methods

### Chemicals

DMSO, β-NF, DMBA, TCDD, paclitaxel*,* doxorubicin, and doxycycline were obtained from Sigma-Aldrich (St. Louis, MO). FICZ, CH223191, and CB7993113 were provided by Dr. M. Pollastri (Northeastern University).

### Cell line acquisition, cell culture, and media

Hs578T and MCF-10F cells were purchased from ATCC and cultured according to ATCC recommendations (ATCC, Manassas, VA). SUM149 cells were a generous gift from Dr. Stephen Ethier (Wayne State University, Detroit, MI). SUM149 cells were maintained in F-12 K Medium (Mediatech, Herndon, VA) containing 5 % FBS (Sigma-Aldrich), 0.5 μg/mL hydrocortisone (Sigma-Aldrich), 2 mM L-glutamine (Mediatech), 100 IU penicillin/100 μg/mL streptomycin (Mediatech), 10 μg/mL insulin (Sigma-Aldrich), and 5 μg/mL Plasmocin (Invivogen, San Diego, CA).

### Inducible, stable *Ahr-*specific shRNA cells

Doxycycline (dox)-inducible *TurboRFP-shAhr* TRIPZ lentiviral vectors (Open Biosystems, Huntsville, AL) were used to make viral transduction particles. Hs578T and SUM149 cells were transduced at optimal MOIs of 25 and 50, respectively, in medium containing hexadimethyrine bromide (8 μM g/mL polybrene; Sigma-Aldrich). Transduced cells were maintained in 1.5 μg/mL puromycin (Invitrogen, Grand Island, NY). RFP expression was maximal 48 hours after dox treatment (1.5 μg/mL) of transduced cells. For in vivo experiments, two different inducible shAHR plasmids were constructed and used to generate two independent doxycycline-inducible shAHR-expressing SUM149 lines.

### ALDEFLUOR™ staining

Cells were dosed with 0.5 μM FICZ, 1 μM β-NF, 10 μM CH223191, 10 μM CB7993113, 1 nM TCDD, 1 μM DMBA, 1.5 μg/mL dox, vehicle (0.1 % DMSO), and/or left untreated every 24 hours. After 48 hours, ALDEFLUOR™ assays were performed according to the manufacturer’s instructions (Stem Cell Technologies, Vancouver, Canada). Briefly, cells (10^6^ cells/mL) were treated with 5 μL/mL ALDEFLUOR^TM^ substrate in 1 mL of ALDEFLUOR^TM^ buffer. Negative controls were treated with both ALDEFLUOR^TM^ substrate and 50 mmol/L diethylaminobenzaldehyde (DEAB), an ALDH-specific inhibitor. Samples were incubated for 35 minutes at 37 °C in the dark. After 35 minutes, cells were centrifuged, the supernatant was removed and the remaining pellet was suspended in ice-cold ALDEFLUOR^TM^ buffer and kept on ice. Before samples were read on the flow cytometer, propidium iodine was added (1.5 μg/mL) to quantify viability (propidium iodine was not used on TurboRFP-shAhr transduced cells due to overlapping emissions). Cells were immediately assayed with an LSRII flow cytometer (Becton Dickinson Biosciences, San Jose, CA) using DEAB controls as baselines to gate ALDH^high^ and ALDH^low^ cell populations. ALDH^high^ and ALDH^low^ cells were sorted on a MoFlo Legacy (Beckman Coulter, Indianapolis, IN). All flow cytometry data was analyzed using Flowjo software (Ashland, OR) according to Stem Cell Technologies’ manufacturer instructions. Briefly, DEAB-treated control samples were used to make ALDH^high^ and ALDH^low^ gates as pictured in Fig. 1. The percent of cells that fell into each gate was then quantified as ALDH^high^ or ALDH^low^ subsets.

### Western blotting

Cells were lysed and protein extracted using NE-PER Nuclear and Cytoplasmic Extraction Reagents (ThermoFisher Scientific, Grand Island, NY), according to manufacturer instructions. Protein concentration was then quantified via a Bradford protein assay. Equal amounts of protein (40 μg) were subjected to 10 % SDS-PAGE and then transferred to a nitrocellulose membrane. Non-specific binding sites were blocked with blocking buffer containing Tris-buffered saline and 0.1 % Tween-20 with 5 % nonfat milk powder for 1 hour at room temperature, and the blot was incubated with specific antibody in blocking buffer (SOX2, Lamin A/C and α-Tubulin antibody in 1:1000 dilution, respectively) at 4 °C overnight. After washing, the blot was incubated with an appropriate secondary antibody conjugated with horseradish peroxidase for 1 hour at room temperature. After washing, the detection was performed using the enhanced chemiluminescence system. The antibody of SOX2 was purchased from Cell Signaling Technology (Danvers, MA; Cat #: 2748), Lamin A/C from Cell Signaling Technology (Cat #: 2032), and α-Tubulin from EMD Millipore (Billerica, MA; Cat #: CP06). Image J (National Institutes of Health, Bethesda, MD) was used to perform densitometry analysis. Fold-change from naïve is presented following normalization to loading control (Lamin A/C for nuclear extract and α-tubulin for cytoplasmic extract).

### Tumorsphere formation

Cells were treated as above. After 48 hours, cells were harvested, dosed, and 3 × 10^3^ cells plated in complete MammoCult Medium (STEMCELL Technologies) containing 0.5 μM hydrocortisone, 2 mM L-glutamine, 100 IU penicillin/100 μM g/mL streptomycin, and 1 % methylcellulose (Sigma Aldrich) in ultra-low adherent 24-well plates (Corning Inc.). Colonies were quantified with a Celigo S Imaging Cytometer (Brooks Automation, Chelmsford, MA) after 8 days. For secondary sphere formation, tumorspheres were mechanically and enzymatically dissociated into a single cell suspension, re-dosed, re-plated, and imaged as above.

### RT-qPCR

mRNA was extracted using RNeasy® Plus Mini Kit (Qiagen, Valencia, CA) and cDNA prepared using the GoScript™ Reverse Transcription System (Promega, Madison, WI) with a 1:1 mixture of random and Oligo (dT)_15_ primers according to manufacturer’s instructions. All RT-qPCR reactions were performed using the GoTaq® RT-qPCR Master Mix System (Promega). Validated primers were purchased from Qiagen Inc. (Valencia, CA): human *Cyp1b1* – QT00209496, *Cyp1a1 – QT00012341*, *Twist1* – QT00011956, *Snai1* – QT00010010, *Snai2* – QT00044128, *VIM* – QT00095795, *Twist2* – QT02454004, *FN1* – QT00038024, *Notch1* – QT01005109, *Notch2* – QT00072212, *Aldh1a1* – QT00013286, *Aldh1a3* – QT00077588, *Pou5f1* – QT00210840, *Sox* – QT00237601, *Nanog* – QT01844808, *Dppa3* – QT01667197, *Msi1* – QT00025389, Human *Bmi1* – QT00052654, *Tgfb1* – QT00000728, *Ahr* – QT02422938, and *Gapdh* – QT01192646. RT-qPCR reactions were performed using a 7900HT Fast Real-Time PCR instrument (Applied Biosystems, Carlsbad, CA), with hot-start activation at 95 °C for 2 min, 40 cycles of denaturation (95 °C for 15 sec), and annealing/extension (55 °C for 60 sec). Relative gene expression was determined using the Pfaffl method [103] and the threshold value for *Gapdh* mRNA was used for normalization.

### Chromatin immunoprecipitation (ChIP) assay

ChIP studies were performed using an AHR-specific antibody (ab2769; Abcam, Cambridge, MA) and the ChIP kit (ab500; Abcam) according to the manufacturer’s protocol. Cells were fixed and sonicated to produce fragments averaging 500 bp. Following immunoprecipitation with AHR-specific antibody or normal mouse IgG (Santa Cruz Biotechnology, Dallas, TX), DNA was purified and amplified using the following primers: *Cyp1b1* primer: 5’-GTTTGGCGCTGGGTTAC-3’ and 5’-AGGTCGGAGCTGACTCTCT-3’ [104], *Sox2* primer: 5’-CTGTGAGAAGGGCGTGAGAG-3’ and 5’- AAACAGCCAGTGCAGGAGTT-3’. The relative DNA amount was calculated using the ΔΔCt method. AHR and IgG control pull-down signal were normalized to input signal.

### Transient transfection

Hs578T or SUM149 cells were co-transfected with the *pGudluc* reporter plasmid (0.5 μg) (generously provided by Dr. M. Denison, UC, Davis), and *CMV-green* (0.1 μg; for normalization) using TransIT-2020 transfection reagent (Mirus, Madison, WI)*.* The transfection medium was replaced after 24 hours. The cells were left untreated or dosed with vehicle (DMSO, 0.1 % final concentration), 0.5 μM FICZ or CH223191 (10 μM), and harvested after 24 hours in Glo Lysis Buffer (Promega, San Luis Obispo, CA). Luciferase activity was determined with the Bright-Glo Luciferase System according to the manufacturer’s instructions (Promega). Luminescence and fluorescence were determined using a Synergy2 multifunction plate reader (Bio-Tek, Winooski, VT).

### Scratch-wound assay

ALDH^high^ and ALDH^low^ cells were sorted and grown to confluence in 12-well plates. A p200 pipet tip was used to make an ‘X’ in each well and non-adherent cells were removed with PBS washes. Media was added and cells treated with vehicle, 10 μM CH223191, 1 nM TCDD, or 0.5 μM FICZ. Media was changed and cells were re-dosed daily. TScratch software (Tobias Gebäck and Martin Schulz, ETH Zürich) was used to quantify the closure of the scratch over time.

### Mouse model

Eight-week old, female non-obese diabetic-severe combined immunodeficiency (NOD/SCID) mice were purchased from Jackson Laboratory (Bar Harbor, ME). To determine if the AHR influences this parameter of BCS_L_Cs, two separate in vivo experiments were performed. For both experiments, SUM149 cells that were stably transduced with either of two dox-inducible shAHR were sorted into ALDH^high^ and ALDH^low^ cell populations. For the first in vivo experiment, 3000 ALDH^high^ and ALDH^low^ cells in 100 μL of 50:50 Matrigel/DMEM were injected into the right and left mammary fat pads, respectively, of female NOD/SCID mice. For the second in vivo experiment, titered numbers (2,500, 5,000, or 10,000) of ALDH^high^ and ALDH^low^ cells in 100 μL of 50:50 Matrigel/DMEM were injected into the right and left mammary fat pads, respectively, of female NOD/SCID mice. For both experiments, control mice drank water with 5 % sucrose, while the treated mice were provided with water containing 5 % sucrose and 2 mg/mL doxycycline to induce the *shAHR*. Tumor growth was quantified using Vernier calipers and animals were sacrificed when the total tumor burden reached 15 mm. No metastases were noted at this time. Necropsies were performed to resect the tumors from both sides. RNA was isolated from each of the primary tumors for gene expression analyses for the first in vivo experiment. Animals were housed at the Association for Assessment and Accreditation of Laboratory Animal Care certified Boston University Medical Laboratory Animal Science Center and used in accordance with the NIH Guide for the Care and Use of Laboratory Animals. A Boston University Medical Campus Institutional Animal Care and Use Committee approved protocol and National Institutes of Health Guide for the Care and Use of laboratory Animals were followed.

### Immunofluorescence

Hs578T and SUM149 cells were grown overnight on glass cover slips. Upon harvest, cells were washed with cold PBS, fixed with 4 % fresh paraformaldehyde for 10 minutes, permeabilized in 0.5 % Triton X-100 for 10 minutes, and blocked with 2 % BSA overnight. Cells were incubated with anti-AHR antibody H-211 (Santa Cruz Biotechnology, Dallas, TX) for 2 hours, washed in PBS and incubated with Alexa Fluor® 594 conjugated anti-rabbit IgG (Molecular Probes, Eugene, OR) for 60 minutes. Cover slips were washed and mounted on slides with ProLong® Gold Antifade Reagent (Life Technologies, Carlsbad, CA). Photomicrography was performed with a Nikon Deconvolution Wide-Field Epifluorescence Microscope using NIS Elements software. No background fluorescence was detectable in samples treated with the secondary antibody alone.

### Immunohistochemistry

Immunohistochemistry was performed on slides of paraffin-embedded, 5 μm-thick sections of breast invasive ductal carcinoma in a tissue microarray (US Biomax, Inc., Rockvilla, MD) by standard protocol on an intelliPATH Automated Slide Staining System from Biocare Medical (Concord, CA). Briefly, the slides were heated for 15 minutes at 60 °C followed by deparaffinization starting with xylene and rehydrated through graded alcohols to distilled water. Antigen-retrieval was then performed using Diva Decloaker (Biocare Medical) reagent at 100 °C for 35 minutes, and then at 85 °C for 10 minutes. Slides were incubated with Biocare Medical Peroxidase 1 solution for 10 minutes at room temperature, washed with TBST, blocked with Biocare Medical Background Sniper for 30 minutes and washed. Primary AHR-specific antibody (clone H-211, 1:50 dilution, Santa Cruz Biotechnology) was diluted in Biocare Medical Da Vinci Green Diluent and incubated for 2 hours at room temperature followed by washing in TBST. Incubation in Biocare Medical Mach 4 Universal HRP Polymer was then performed for 30 minutes followed by washing in TBST. DAB was diluted in DAB substrate buffer and applied to slides for 5 minutes followed by washing in deionized-H_2_O. A light hematoxylin stain was applied, the slides were dehydrated, air dried, and mounted, using EcoMount and a coverslip. Microphotography was performed with an Olympus Upright Microscope using QCapture software. No background stain was detectable in the absence of AHR-specific antibody.

### Statistical analyses

Statistical analyses were performed with Prism (GraphPad Software, La Jolla, CA) or StatPlus (Alexandria, VA) unless otherwise noted. Data are presented as mean ± standard error where applicable. One-way analysis of variants (ANOVAs; simple) were used to determine significance. For experiments measuring relative fold-changes in gene expression (determined using the Pfaffl method [103] with *Gapdh* mRNA used for normalization), statistical analyses were performed using SAS v9.3. For comparisons of fold-change in vehicle-treated ALDH^high^ versus vehicle-treated ALDH^low^, and FICZ-treated ALDH^low^ versus vehicle-treated ALDH^low^ cells, *Gapdh*-normalized expression levels were normalized to expression levels in vehicle-treated ALDH^low^ cells. For comparisons of fold-change with FICZ-treated ALDH^high^ versus vehicle-treated ALDH^high^ cells, *Gapdh*-normalized expression levels were normalized to expression levels in vehicle-treated ALDH^high^ cells. Statistical significance was determined with the Wilcoxon rank sum test. Statistical analyses of the mouse model compared the average rate of change over time between groups using a random effects model with a random intercept for each mouse. Day 22 was used as the starting (baseline) value to calculate the rate of change. All mouse analyses were performed using SAS v9.3 using a 0.05 level of significance.

### Availability of supporting data

The data sets supporting the results of this article are included within the article and its additional files (Additional file 7, excel document). For further information, please contact the corresponding author.

## Additional files

Additional file 1: Additional Figure S1. Nuclear AHR staining in triple negative breast cancer cell lines and primary breast cancers. (A) AHR immunofluorescence staining in SUM149 (left) and Hs578T (right) triple negative breast cancer cell lines are shown (Top: bright field in black and white; Bottom: Alexafluor fluorescence in black and white). (B) AHR-specific staining in tissue from two representative human breast cancers from a total of 50 samples is shown (Top: normal breast control, Middle: Her 2^+^, invasive ductal carcinoma; Bottom: ER^−^/PR^−^/Her2^−^ invasive ductal carcinoma). (PDF 10468 kb) Additional file 2: Additional Figure S2. AHR modulation alters ALDH activity in ER^−^, immortalized MCF-10 F epithelial cells. (A) Representative flow cytometry dot plots of ALDEFLUOR™ staining of MCF10F cells treated for 48 hours with vehicle, 10 μM CH223191, or 0.5 μM FICZ. Regions representing ALDH^high^ cells were drawn based on the signal generated in the presence of DEAB. (B) MCF-10 F cells were treated as in (A) and assayed for the percentage of ALDH^high^ cells. Data from four experiments were normalized to results obtained with naive cells (mean baseline = 0.2 % ALDH^high^ cells) and presented as mean fold-change from naive ± standard error. Asterisks indicate a significant decrease in the percentage of ALDH^high^ cells, **P* <0.05, ***P* <0.005. A cross indicates a significant increase in ALDH^high^ cells, ^+^ *P* <0.05. (PDF 97 kb) Additional file 3: Additional Figure S3. Limited plasticity of SUM149 with regard to ALDH expression. SUM149 cells were sorted into ALDH^high^ and ALDH^low^ populations and treated for 96 hours with vehicle or 1 μM CH223191. Every 24 hours, cells were assayed for the percent of (A) ALDH^high^ and (B) ALDH^low^ cells to determine how quickly cells revert to the baseline ALDH^high^ and ALDH^low^ levels. Data are presented as the means from 3 (72 and 96 hours) or 4 (0, 24 and 48 hours) experiments ± standard errors. (PDF 48 kb) Additional file 4: Additional Figure S4. AHR agonists accelerate migration of SUM149 cells. (A) Representative images of cell migration at 24 and 48 hours after SUM149 cells were sorted into the ALDH^high^ population, cultured to confluence, scratched, and treated with vehicle, 0.5 μM FICZ or 1 nM TCDD. Data are representative of five independent experiments. Black lines indicate the borders of the original scratch. (B) ALDH^high^ SUM149 cells were treated as in (A) and the percent exposed area was quantified at 24 and 48 hours. Data from five experiments were normalized to results obtained with naive cells and presented as the mean percent exposed area ± standard error. Asterisks indicate a significant decrease in exposed area, **P* <0.05, ***P* <0.01. (PDF 6744 kb) Additional file 5: Additional Figure S5.
*Ahr* and *Cyp1b1* expression correlate with expression of stem cell- and migration/invasion-associated genes in the CCLE database. The gene set enrichment analysis (GSEA) tool (http://www.broad.mit.edu/gsea) was used to rank genes from the cancer cell line encyclopedia (CCLE) dataset [66] based on the correlation of their expression profiles with (A) *Ahr* and (B) *Cyp1b1* expression. Considering stem cell- and migration/invasion-associated genes present in the CCLE microarray data, their position in the ranked list (represented with vertical black lines in the panels) incremented the enrichment score statistic (ES, plotted in green). A significant positive correlation between *Ahr* or *Cyp1b1* and the gene set was demonstrated by GSEA (*P* = 0.025 and 0.021, respectively), with *Msi1* showing the lowest correlation value in both analyses. (PDF 99 kb) Additional file 6: Additional Figure S6.
*Ahr* and *Cyp1b1* expression correlate with expression of stem cell- and migration/invasion-associated genes in the TCGA database. Expression of genes from the cancer genome atlas (TCGA) dataset [68] were ranked based on the correlation of their expression profiles with *Ahr* (A) and *Cyp1b1* (B) expression. The enrichment score (in green) was computed by considering the position of the stem cell- and migration/invasion-associated genes in the ranked list obtained from the TCGA RNA-Seq data. A significant positive correlation between expression of the putative AHR target genes and *Ahr* or *Cyp1b1* expression (*P* = 0.047, *P* = 0.0001, respectively) was demonstrated. The ranking of *Msi1* demonstrated a low correlation with both *Ahr* and *Cyp1b1* expression. (PDF 100 kb) Additional file 7: The data sets supporting the results of each of the figures in this article are included within this excel document. For further information, please contact the corresponding author. (XLSX 81 kb)
