# Supplementary figures and images for: The role of the aryl hydrocarbon receptor in the development of cells with the molecular and functional characteristics of cancer stem-like cells
Source: BMC Biol. 2016 Mar 16;14:20. doi: 10.1186/s12915-016-0240-y (PMC4794823; doi:10.1186/s12915-016-0240-y)

**A****SUM149**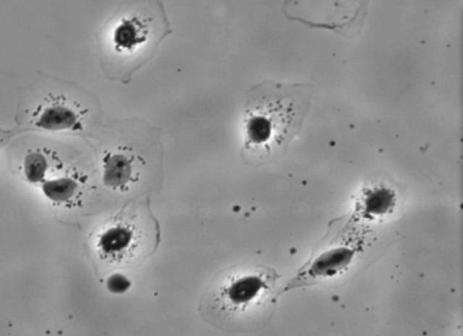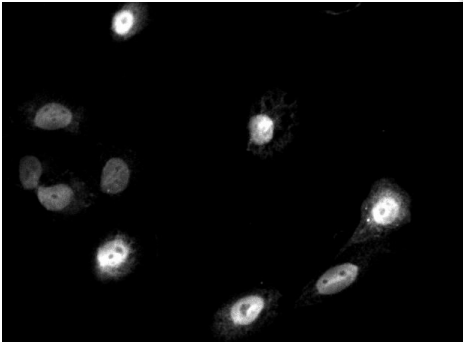**Hs578T**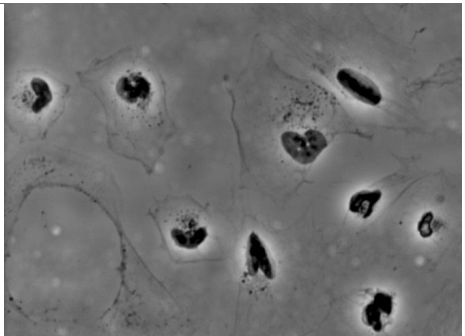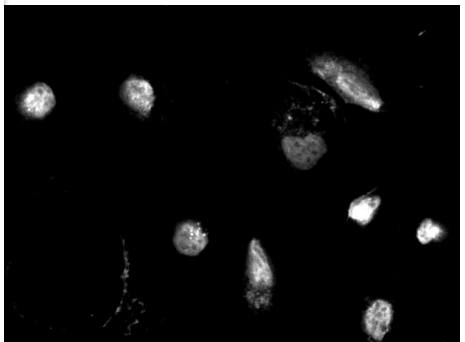**B****Primary**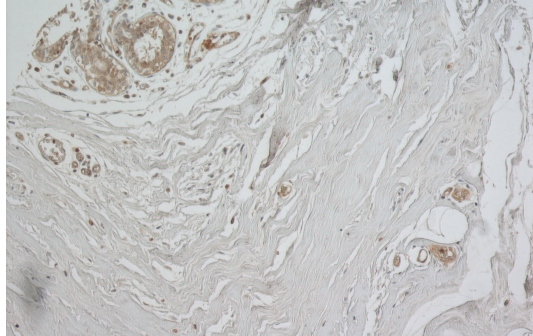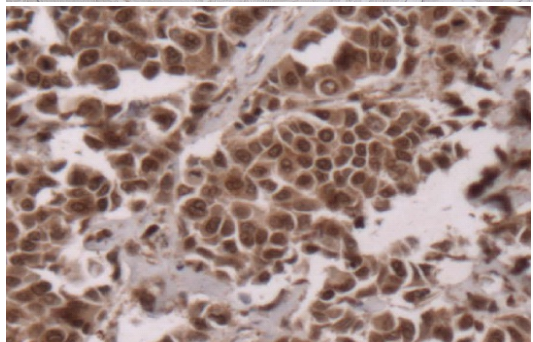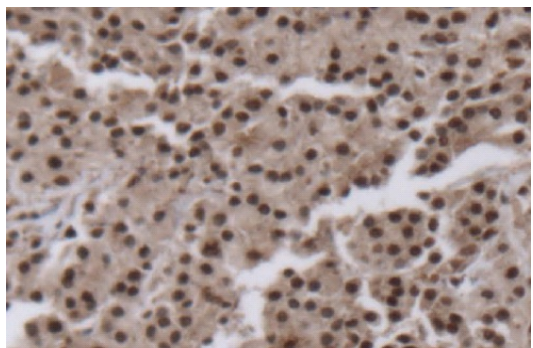

Supplement: Additional file 1: Additional Figure S1. — Nuclear AHR staining in triple negative breast cancer cell lines and primary breast cancers. (A) AHR immunofluorescence staining in SUM149 (left) and Hs578T (right) triple negative breast cancer cell lines are shown (Top: bright field in black and white; Bottom: Alexafluor fluorescence in black and white). (B) AHR-specific staining in tissue from two representative human breast cancers from a total of 50 samples is shown (Top: normal breast control, Middle: Her 2+, invasive ductal carcinoma; Bottom: ER−/PR−/Her2− invasive ductal carcinoma). (PDF 10468 kb) [file 12915_2016_240_MOESM1_ESM.pdf]

A

Vehicle

CH223191

FICZ

FICZ+CH223191

SSC

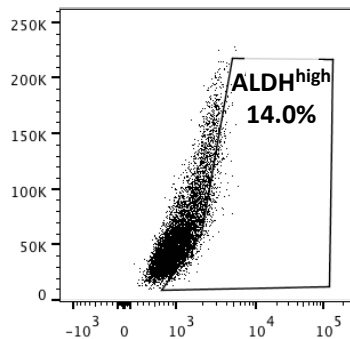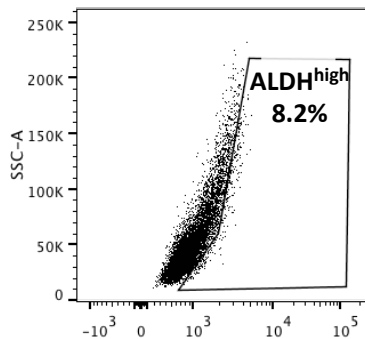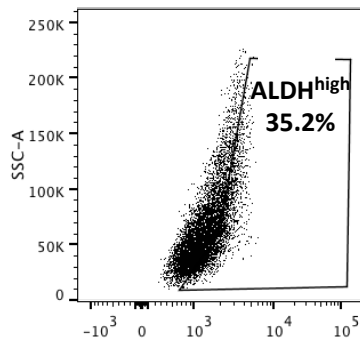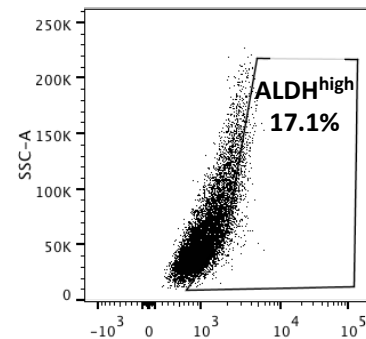

ALDH

%ALDH<sup>high</sup> Fold-Change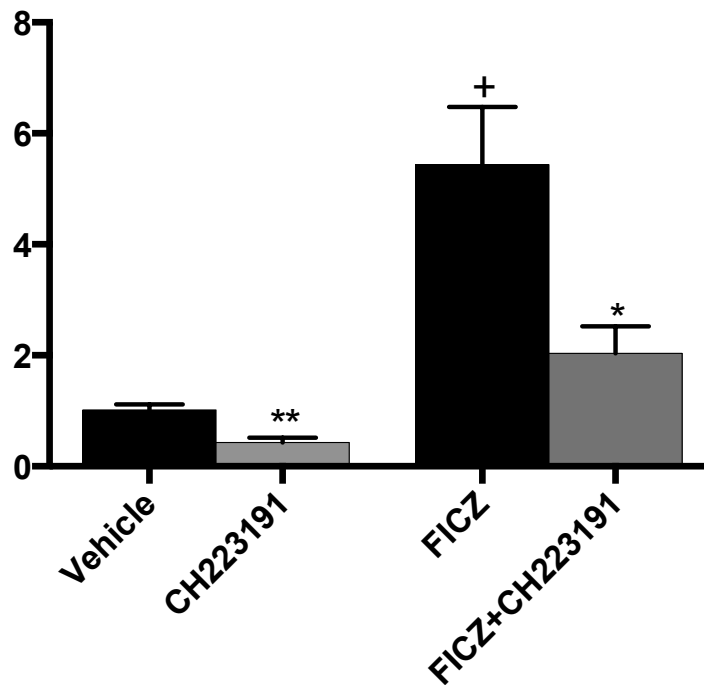

Supplement: Additional file 2: Additional Figure S2. — AHR modulation alters ALDH activity in ER−, immortalized MCF-10 F epithelial cells. (A) Representative flow cytometry dot plots of ALDEFLUOR™ staining of MCF10F cells treated for 48 hours with vehicle, 10 μM CH223191, or 0.5 μM FICZ. Regions representing ALDHhigh cells were drawn based on the signal generated in the presence of DEAB. (B) MCF-10 F cells were treated as in (A) and assayed for the percentage of ALDHhigh cells. Data from four experiments were normalized to results obtained with naive cells (mean baseline = 0.2 % ALDHhigh cells) and presented as mean fold-change from naive ± standard error. Asterisks indicate a significant decrease in the percentage of ALDHhigh cells, *P <0.05, **P <0.005. A cross indicates a significant increase in ALDHhigh cells, + P <0.05. (PDF 97 kb) [file 12915_2016_240_MOESM2_ESM.pdf]

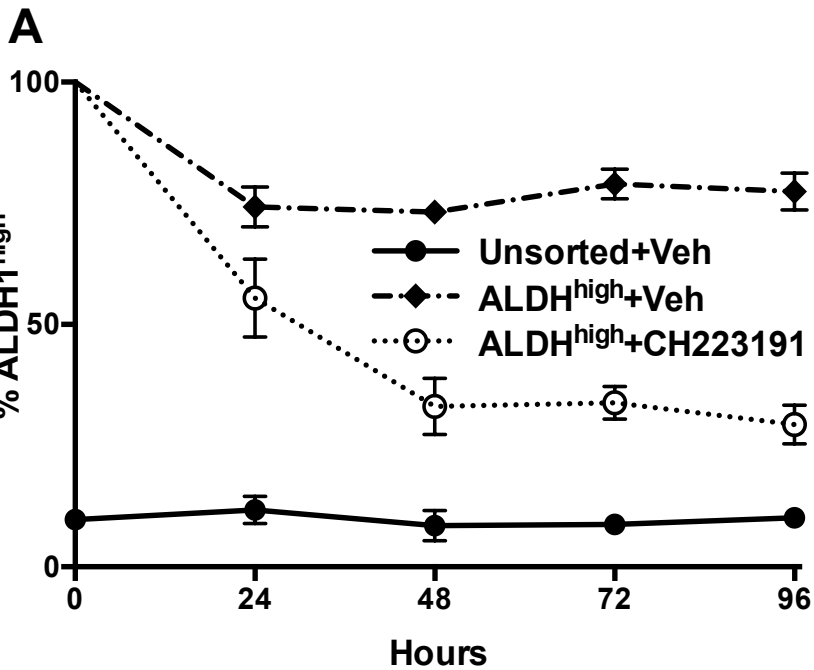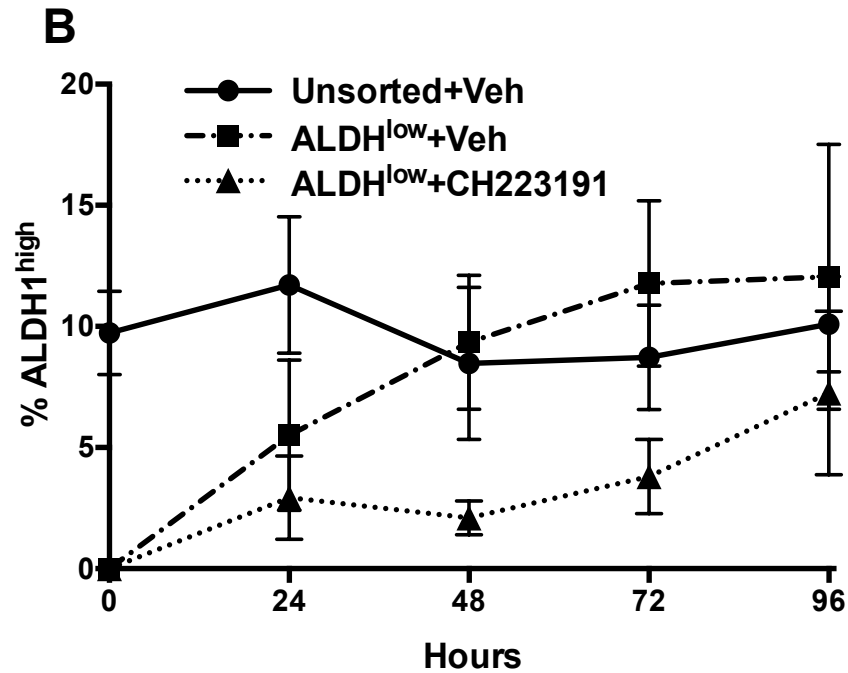

Supplement: Additional file 3: Additional Figure S3. — Limited plasticity of SUM149 with regard to ALDH expression. SUM149 cells were sorted into ALDHhigh and ALDHlow populations and treated for 96 hours with vehicle or 1 μM CH223191. Every 24 hours, cells were assayed for the percent of (A) ALDHhigh and (B) ALDHlow cells to determine how quickly cells revert to the baseline ALDHhigh and ALDHlow levels. Data are presented as the means from 3 (72 and 96 hours) or 4 (0, 24 and 48 hours) experiments ± standard errors. (PDF 48 kb) [file 12915_2016_240_MOESM3_ESM.pdf]

ALDH<sup>high</sup> VehicleALDH<sup>high</sup> FICZALDH<sup>high</sup> TCDD

A

24  
hours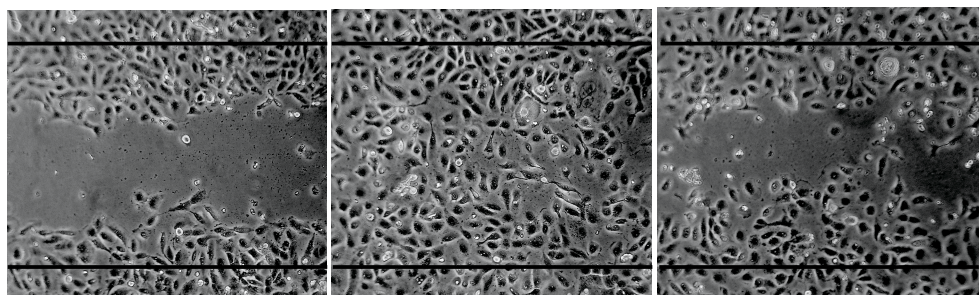48  
hours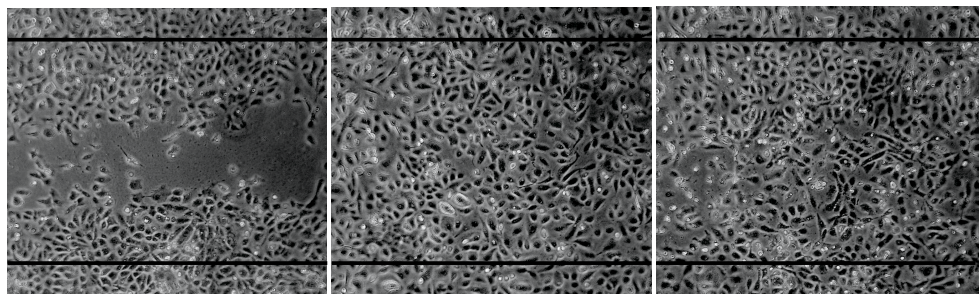

B

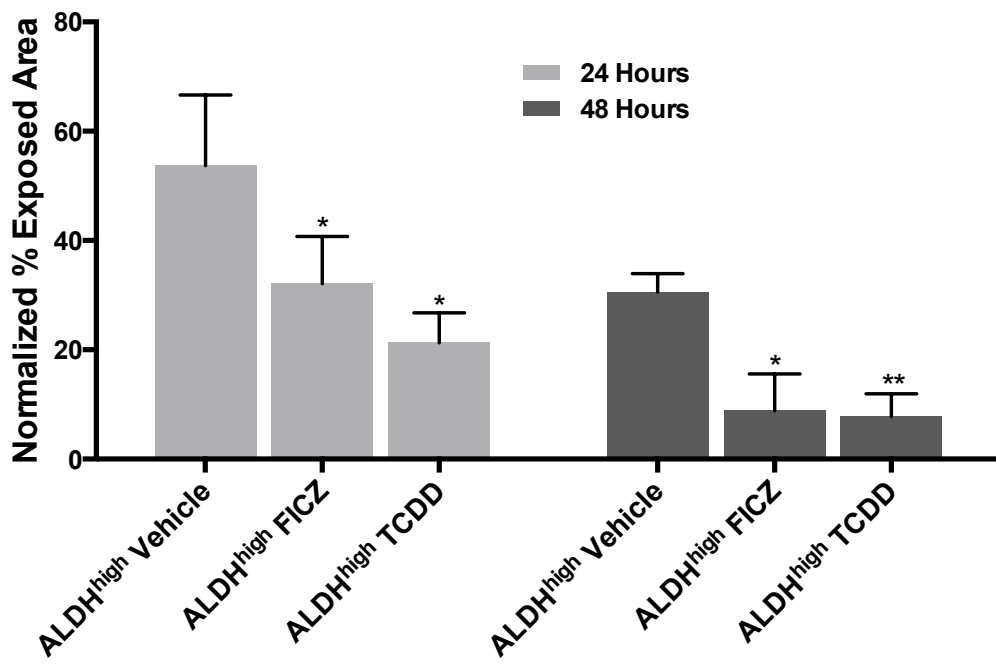

Supplement: Additional file 4: Additional Figure S4. — AHR agonists accelerate migration of SUM149 cells. (A) Representative images of cell migration at 24 and 48 hours after SUM149 cells were sorted into the ALDHhigh population, cultured to confluence, scratched, and treated with vehicle, 0.5 μM FICZ or 1 nM TCDD. Data are representative of five independent experiments. Black lines indicate the borders of the original scratch. (B) ALDHhigh SUM149 cells were treated as in (A) and the percent exposed area was quantified at 24 and 48 hours. Data from five experiments were normalized to results obtained with naive cells and presented as the mean percent exposed area ± standard error. Asterisks indicate a significant decrease in exposed area, *P <0.05, **P <0.01. (PDF 6744 kb) [file 12915_2016_240_MOESM4_ESM.pdf]

A

# *Ahr* vs Stem Cell-Associated Gene Set (CCLE)

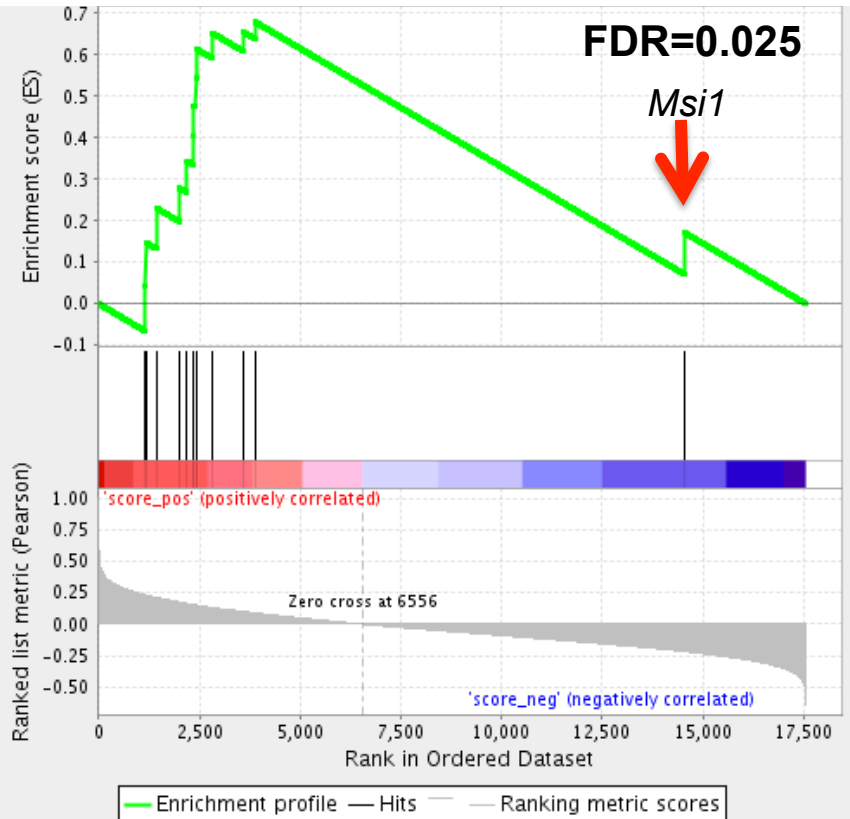

B

# *Cyp1b1* vs Stem Cell-Associated Gene Set (CCLE)

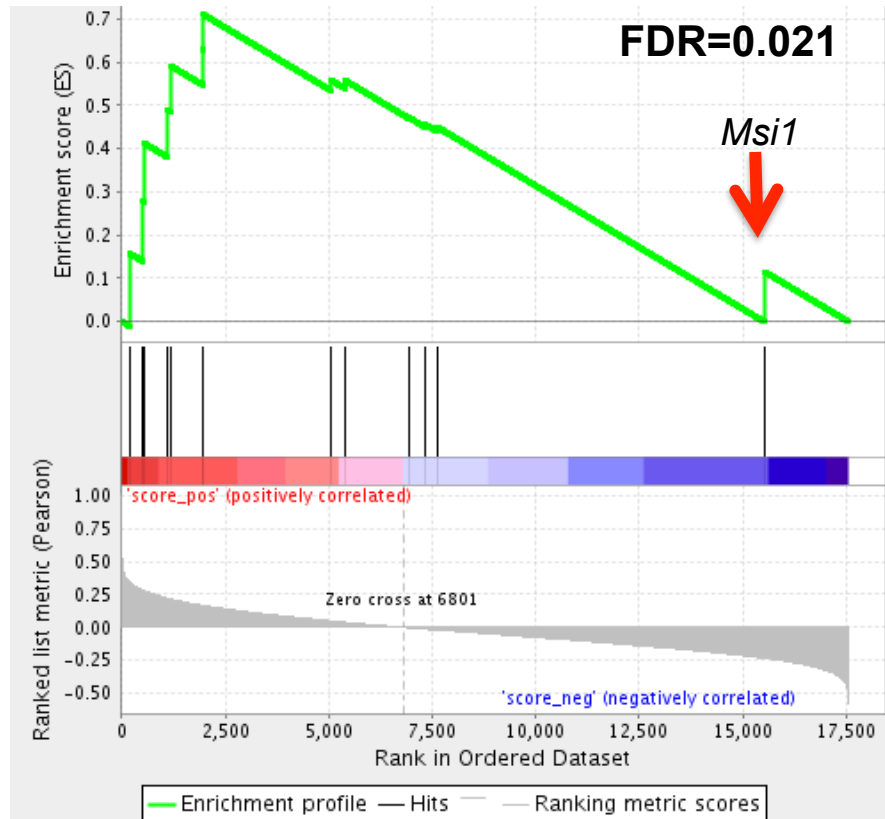

Supplement: Additional file 5: Additional Figure S5. — Ahr and Cyp1b1 expression correlate with expression of stem cell- and migration/invasion-associated genes in the CCLE database. The gene set enrichment analysis (GSEA) tool (http://www.broad.mit.edu/gsea) was used to rank genes from the cancer cell line encyclopedia (CCLE) dataset [66] based on the correlation of their expression profiles with (A) Ahr and (B) Cyp1b1 expression. Considering stem cell- and migration/invasion-associated genes present in the CCLE microarray data, their position in the ranked list (represented with vertical black lines in the panels) incremented the enrichment score statistic (ES, plotted in green). A significant positive correlation between Ahr or Cyp1b1 and the gene set was demonstrated by GSEA (P = 0.025 and 0.021, respectively), with Msi1 showing the lowest correlation value in both analyses. (PDF 99 kb) [file 12915_2016_240_MOESM5_ESM.pdf]

A

# *Ahr* vs Stem Cell-Associated Gene Set (TCGA)

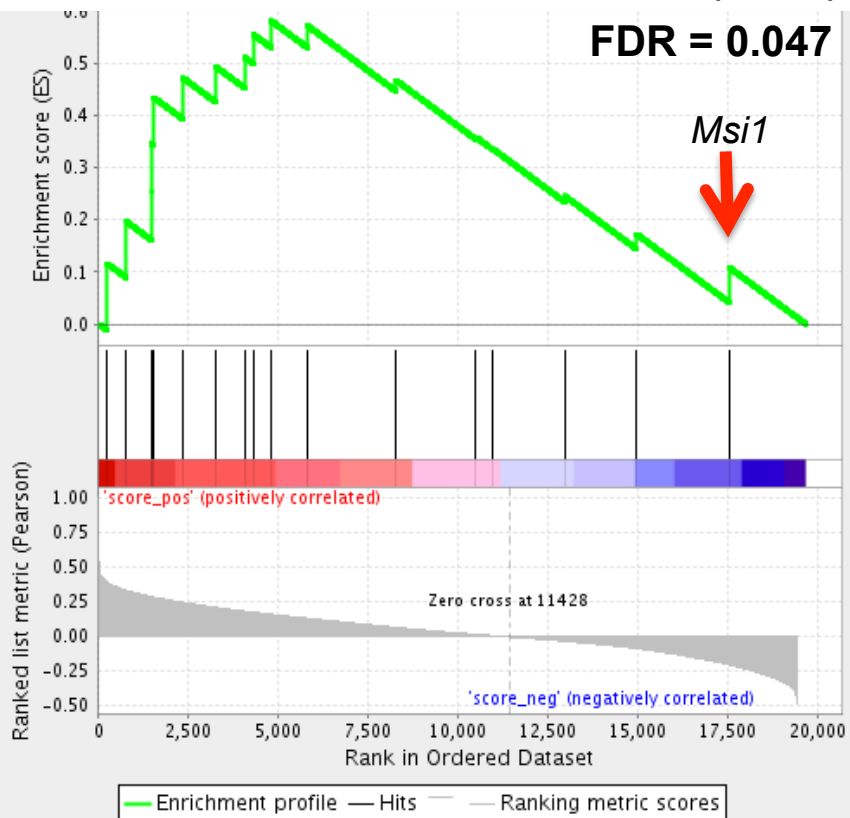

B

# *Cyp1b1* vs Stem Cell-Associated Gene Set (TCGA)

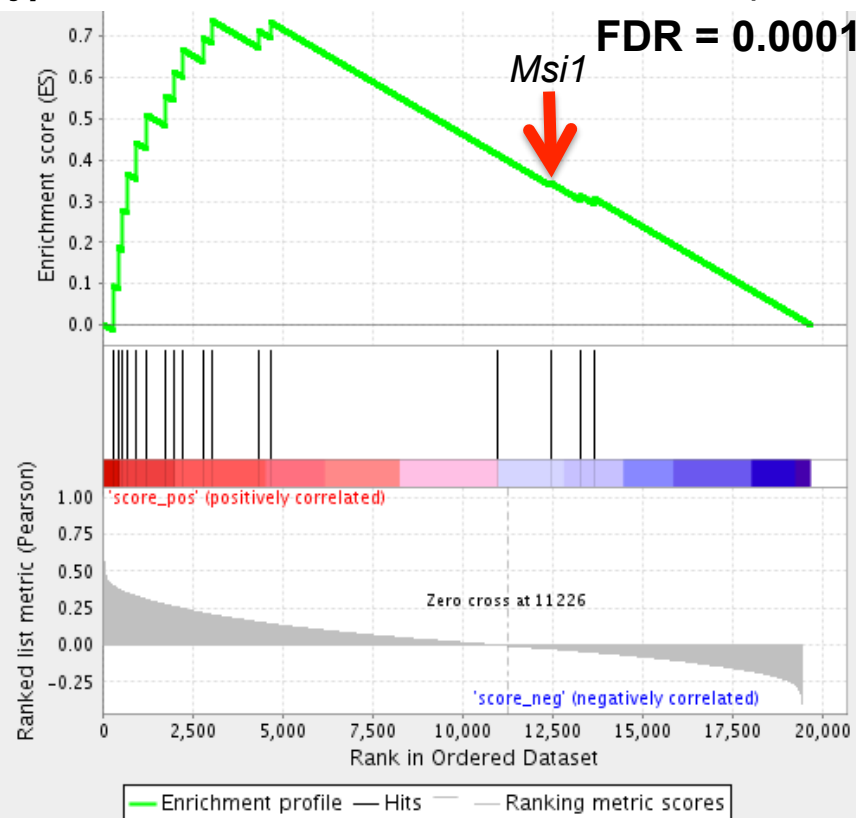

Supplement: Additional file 6: Additional Figure S6. — Ahr and Cyp1b1 expression correlate with expression of stem cell- and migration/invasion-associated genes in the TCGA database. Expression of genes from the cancer genome atlas (TCGA) dataset [68] were ranked based on the correlation of their expression profiles with Ahr (A) and Cyp1b1 (B) expression. The enrichment score (in green) was computed by considering the position of the stem cell- and migration/invasion-associated genes in the ranked list obtained from the TCGA RNA-Seq data. A significant positive correlation between expression of the putative AHR target genes and Ahr or Cyp1b1 expression (P = 0.047, P = 0.0001, respectively) was demonstrated. The ranking of Msi1 demonstrated a low correlation with both Ahr and Cyp1b1 expression. (PDF 100 kb) [file 12915_2016_240_MOESM6_ESM.pdf]
